# Targeting parvalbumin promotes M2 macrophage polarization and energy expenditure in mice

**DOI:** 10.1038/s41467-022-30757-y

**Published:** 2022-06-08

**Authors:** Shaojian Lin, Anke Zhang, Ling Yuan, Yufan Wang, Chuan Zhang, Junkun Jiang, Houshi Xu, Huiwen Yuan, Hui Yao, Qianying Zhang, Yong Zhang, Meiqing Lou, Ping Wang, Zhen-Ning Zhang, Bing Luan

**Affiliations:** 1 Department of Endocrinology, Tongji Hospital Affiliated to Tongji University, School of Medicine, Tongji University, Shanghai, China; 2 Department of Neurosurgery, Ruijin Hospital, Shanghai Jiao Tong University School of Medicine, Shanghai, China; 3 Department of Neurosurgery, Second Affiliated Hospital, School of Medicine, Zhejiang University, Hangzhou, Zhejiang Province China; 4 Department of Endocrinology and Metabolism, Shanghai General Hospital, Shanghai Jiao Tong University, Shanghai, China; 5 Department of Neurosurgery, Shanghai General Hospital, Shanghai Jiao Tong University School of Medicine, Shanghai, China; 6 The State Key Laboratory of Medical Molecular Biology, Institute of Basic Medical Sciences, Chinese Academy of Medical Sciences and School of Basic Medicine, Peking Union Medical College, Beijing, China; 7 Tongji University Cancer Center, Shanghai Tenth People’s Hospital, School of Medicine, Tongji University, Shanghai, China; 8 Translational Medical Center for Stem Cell Therapy & Institute for Regenerative Medicine, Shanghai East Hospital, School of Life Sciences and Technology, Tongji University, Shanghai, China

**Keywords:** Monocytes and macrophages, Inflammation, Obesity, Proteomics

## Abstract

Exercise benefits M2 macrophage polarization, energy homeostasis and protects against obesity partially through exercise-induced circulating factors. Here, by unbiased quantitative proteomics on serum samples from sedentary and exercised mice, we identify parvalbumin as a circulating factor suppressed by exercise. Parvalbumin functions as a non-competitive CSF1R antagonist to inhibit M2 macrophage activation and energy expenditure in adipose tissue. More importantly, serum concentrations of parvalbumin positively correlate with obesity in mouse and human, while treating mice with a recombinant parvalbumin blocker prevents its interaction with CSF1R and promotes M2 macrophage polarization and ameliorates diet-induced obesity. Thus, although further studies are required to assess the significance of parvalbumin in mediating the effects of exercise, our results implicate parvalbumin as a potential therapeutic strategy against obesity in mice.

## Abstract (web summary)

Exercise increases energy expenditure and suppresses obesity, but the effector mechanisms are not still unclear. Here the authors profile serum proteomics in exercised mice to find reduced parvalbumin levels that correlate with increased M2 macrophage and suppressed diet-induced obesity to hint parvalbumin as a potential therapy target against obesity.

## Introduction

The worldwide prevalence of obesity is mainly caused by the imbalance between energy intake and energy expenditure. Brown and beige adipose tissues have become unique targets for the treatment of obesity since the discovery of their importance in promoting energy expenditure by non-shivering thermogenesis^1^. Accumulated evidence suggests that activation of brown and beige adipose tissue thermogenesis ameliorates obesity and related insulin resistance; ablation of brown or beige fat deteriorates its progress^2–5^. The ability of these depots to dissipate energy relies largely on uncoupling protein 1 (UCP1)^6^, although UCP1-independent thermogenic pathways have also been characterized in brown adipocytes^7,8^.

While catecholamine released by cold stimulated sympathetic nerve system induces UCP1 expression in brown and beige adipose tissues, alternatively activated M2 macrophages also contribute to brown and beige adipose tissues thermogenesis, although the mechanisms are still under debate (9–11). M2 macrophages are reprogrammed by cytokines such as interleukin4 (IL4) or IL13. In addition, growth factors such as Macrophage colony-stimulating factor (MCSF) or Granulocyte macrophage colony-stimulating factor (GMCSF) also regulates M2 macrophage polarization^9,10^. Circulating factors that modulate M2 macrophage polarization in adipose play pivotal roles in brown and beige thermogenic regulation. Numerous factors such as CXCL14, Adiponectin, FGF21 and Succinate have been identified and proved to participate in this regulation^11–14^. Thus, to identify new M2 macrophage modulators may represent a promising strategy for brown and beige thermogenic regulation and the treatment of obesity.

Exercise benefits energy homeostasis partially by increasing energy expenditure in mammals and exercise-induced hormones have emerged as a new class of M2 macrophage polarization and thermogenesis regulators. Irisin, meteorin-like (Metrnl) and β-aminoisobutyric acid (BAIBA) released from muscle promotes thermogenesis in adipose tissue^15–17^. 12,13-Dihydroxyoctadecaenoic acid (12,13-diHOME), an exercise-induced lipokine secreted from BAT induces fuel uptake in BAT and supports thermogenesis^18^.

Here, we identify parvalbumin as an exercise-repressed circulating factor in mice and parvalbumin treatment suppresses M2 macrophage activation and thermogenesis. Moreover, targeting parvalbumin by a synthetic blocker that blocks the interaction between parvalbumin and colony stimulating factor 1 receptor (CSF1R) ameliorates diet-induced obesity in mice. Taken together, this study implicates parvalbumin as a potential therapeutic strategy against obesity, which will need further evaluation in humans.

## Results

### Identification of parvalbumin as an Exercise-suppressed regulator of M2 macrophage

**Fig. 1:**
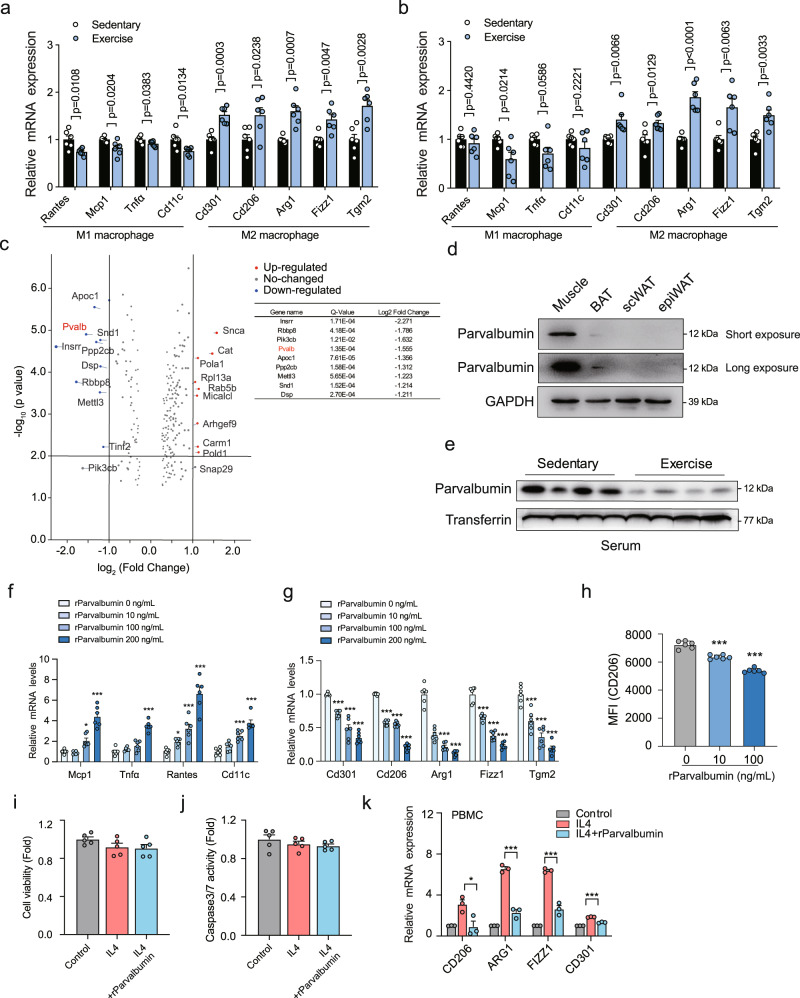
Identification of parvalbumin as an Exercise-suppressed regulator of M2 macrophage. Representative M1 and M2 macrophage marker gene expression in scWAT (**a**) and BAT (**b**) of sedentary and exercised mice. Sedentary, *n* = 6; exercise, *n* = 6. **c** Volcano plot of secreted proteins altered in serum from treadmill-running mice compared with sedentary controls (*n* = 3 with 3 mouse serums per pool). Multiple hypothesis correction were performed on the data and q-value was used to replace p-values. **d** Parvalbumin protein levels in muscle and adipose tissues. **e** Parvalbumin protein levels in serum from exercised or sedentary mice. Representative M1 (**f**) (*P* value: 0.016, <0.0001, <0.0001, 0.0307, <0.0001, <0.0001, 0.0003, <0.0001) and M2 (**g**) (*P* value: ***p < 0.0001) macrophage marker gene expression in BMDM exposed to indicated concentration of parvalbumin (*n* = 6 per group). **h** Flow cytometric analysis of M2 macrophage marker CD206 in BMDM treated with or without parvalbumin (*n* = 6 per group). *P* value: ***p < 0.0001. **i** Cell viability and (**j**) Caspase 3/7 activity in BMDMs treated with or without parvalbumin (100 ng/ml) (*n* = 5 per group). **k** Representative M2 macrophage marker gene expression in human PBMCs treated with or without human parvalbumin (100 ng/ml) (*n* = 3 per group). *P* value: 0.0346, 0.0003, 0.0005, 0.0002. *n* values refer to the number of mice and human serum samples used. Data are represented as mean ± SEM. *P* values were determined by unpaired two-tailed Student’s *t*-test **a**, **b**, **c** and **i**–**k** or one-way ANOVA with Tukey’s post hoc tests (**f**–**h**). *p < 0.05, **p < 0.01, ***p < 0.001. Source data are provided as a Source Data file.

Reports show that exercise leads to increase of M2 macrophages and decrease of M1 macrophages in both brown adipose tissue (BAT) and subcutaneous white adipose tissue (scWAT)^15,19,20^, which prompted us to use this model to identify new circulating factors that could mediate exercise-induced M2 macrophage polarization. We subjected 8-week male mice to treadmill exercise on a motorized, speed-controlled treadmill system (12 m/min, 1 h/day for 6 days/week with a 10% inclination angle) for 6 weeks. 12 h after the last exercise, mice were euthanatized and we were able to confirm that the mRNA levels of mitochondrial adaption-associated genes such as *Pgc1α*, *Atf2*, and *Drp1*^21–23^ were increased in muscle of exercised mice, suggesting the successful establishment of chronic exercise model (Fig. s1a). Meanwhile, M2 macrophage marker genes expression was upregulated while M1 macrophage marker genes expression was downregulated in scWAT and BAT of exercised mice compared with sedentary ones (Fig. 1a, b). We then collected the serum for unbiased quantitative proteomics analyses using the Tandem Mass Tag (TMT) method. In consistency with previous reports, APOC1, APOC4^24^ and Creatine kinase M (CKM)^25^ was decreased, while catalase (CAT)^11^ and Coactivator associated arginine methyltransferase 1 (CARM1)^12^ was increased after exercise in our proteomic screen (Fig. 1c). Among the top 9 proteins significantly decreased by exercise (Fig. 1c), 6 proteins including parvalbumin, Protein phosphatase 2 catalytic subunit beta (PPP2CB), Phosphatidylinositol-4,5-bisphosphate 3-kinase catalytic subunit beta (PIK3CB), Staphylococcal nuclease and tudor domain containing 1 (SND1), Desmoplakin (DSP), and Insulin receptor related receptor (INSRR) have commercial available recombinant proteins and their roles in immune regulation and the underlying mechanisms remain unclear. We used all 6 recombinant human proteins to stimulated human peripheral blood mononuclear cells (PBMCs) together with IL4. As is shown in Fig. s1b, the expression of M2 macrophage marker genes were decreased with the treatment of parvalbumin only, while the stimulation of the other proteins didn’t exert such effect (Fig. s1b).

Parvalbumin, a classical member of the EF-hand superfamily^26,27^, is highly expressed in muscles in mice and is much less expressed in brain, kidney, parathyroid and BAT^28^ (Fig. 1d and Fig. s1c, d). We confirmed that serum levels of parvalbumin were significantly suppressed in exercised mice compared with sedentary mice by immunoblot (Fig. 1e). The identity of the antibody recognized parvalbumin band was validated by mass spectrometry (Fig. s1e, f). Meanwhile, parvalbumin mRNA levels and protein levels were also downregulated in skeletal muscles of exercised mice compared with sedentary mice, while remained unchanged in kidney or parathyroid (Fig. s1g, h). Indeed, we were able to detect parvalbumin from medium of either primary cultured muscle fibres or muscle tissues (Fig. s2a, b). Furthermore, when lentivirus expressing a shRNA targeting parvalbumin (Lenti-shParvalbumin) were locally injected in muscle to silence parvalbumin expression, serum levels of parvalbumin were no longer decreased in exercised mice compared with sedentary mice indicating that muscle is an important source of exercise-regulated parvalbumin secretion (Fig. s2c). Although parvalbumin expression is not limited in muscle in humans (http://biogps.org), the data from GSE 9103 (https://www.ncbi.nlm.nih.gov/geo/query/acc.cgi?acc=GSE9103) indicated downregulated parvalbumin expression in exercised human individuals (Fig. s2d). However, whether parvalbumin secretion from muscle is altered by exercise in humans and its functional relevance to human obesity will need further evaluation.

Parvalbumin is not a typical secretory protein and does not possess a signal peptide, however, we found that parvalbumin could be detected in the small EVs secreted from muscle cells.

The EVs particle size and concentration were measure by transmission electron microscope and nanoparticle tracking analysis (NTA) (Fig. s2e, f). Indeed, when exosome release inhibitor GW4869 was locally injected in muscle for 6 h, small EVs as well as parvalbumin secretion to the serum were abrogated suggesting that parvalbumin could be secreted by muscle through small EVs (Fig. s2g, h).

We next use recombinant parvalbumin protein to further test the role of parvalbumin in M2 macrophage polarization. Indeed, parvalbumin treatment on bone marrow-derived macrophages (BMDMs) greatly inhibited M2 macrophage marker expression but increased M1 macrophage marker expression in a dose-dependent manner (Fig. 1f, g). Flow cytometry analysis of parvalbumin treatment on BMDMs also showed inhibited M2 macrophage marker CD206 positive expression (Fig. 1h), while macrophage proliferation and apoptosis were not influenced (Fig. 1i, j). Furthermore, to gain insight into whether parvalbumin may inhibit markers of M2 macrophages in humans, we obtained human PBMCs from 4 healthy humans and found that recombinant human parvalbumin (100 ng/ml) inhibited IL4-induced M2 macrophage marker expression in these cells (Fig. 1k).

### Circulating parvalbumin suppresses M2 macrophage polarization

**Fig. 2:**
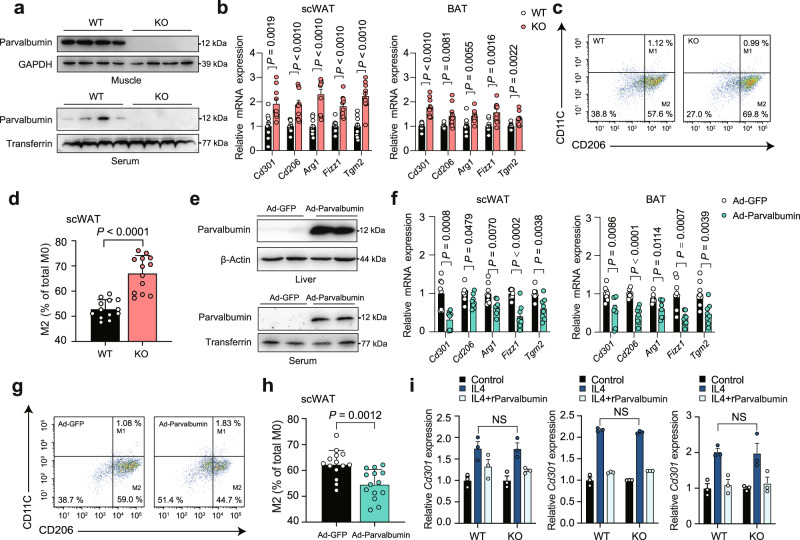
Circulating parvalbumin suppresses M2 macrophage polarization in mice. **a** Parvalbumin protein expression in muscle and serum from WT and parvalbumin KO mice. **b** Representative M2 macrophage marker gene expression in scWAT and BAT of WT and parvalbumin KO mice. WT, *n* = 10; PVALB KO, *n* = 10. **c** Representative flow cytometry plots and (**d**) quantification demonstrate the numbers of M2 macrophages (CD206^+^/CD11C^−^) in the scWAT of WT and parvalbumin KO mice. WT, *n* = 13; PVALB KO, *n* = 13. **e** Male parvalbumin KO mice were intravenously injected with Ad-parvalbumin or Ad-GFP through the tail vein. Parvalbumin protein expression in the liver and serum were examined. **f** Representative M2 macrophage marker gene expression in scWAT and BAT of parvalbumin KO mice injected with Ad-GFP or Ad-parvalbumin. Adv-GFP, *n* = 8; adv-PVALB, *n* = 8. **g** Representative flow cytometry plots and (**h**) quantification demonstrate the numbers of M2 macrophages (CD206^+^/CD11C^−^) in the scWAT of parvalbumin KO mice injected with Ad-GFP and Ad-parvalbumin. Adv-GFP, *n* = 14; Ad-parvalbumin, *n* = 14. **i** Macrophage parvalbumin deficiency had no effect on M2 activation. *Cd301, Cd206*, and *Tgm2* mRNA expression in WT and parvalbumin KO BMDM treated with IL4 and parvalbumin (100 ng/ml) (*n* = 3 per group). Data are represented as mean ± SEM. *P* values were determined by unpaired two-tailed Student’s *t* test (**b**, **d**, **f**, **h**, and **i**). Source data are provided as a Source Data file.

To identify the circulating parvalbumin function, we used the male parvalbumin knockout (KO) mice as a discovery tool. We first examined parvalbumin expression in muscles and serum of KO and WT mice using western blot (Fig. 2a). Next, we use parvalbumin KO mice to further address the role of parvalbumin in M2 macrophage polarization in vivo. Indeed, real-time PCR analyses demonstrated that markers of M2 macrophages, including *Cd301, Cd206, Arg1, Fizz1 and Tgm2* were significantly increased in scWAT and BAT of parvalbumin KO mice (Fig. 2b). Consistently, flow cytometry analysis of the Stromal Vascular Fraction (SVF) from scWAT showed increased composition of M2 macrophages (CD11c-low/CD206-high) in parvalbumin KO mice, suggesting direct modulation of M2 macrophage phenotype by parvalbumin (Fig. 2c, d). To determine if circulating serum parvalbumin contributes to this effect, we performed intravenous injections of adenovirus to deliver parvalbumin constructs (Ad-parvalbumin) to the liver of parvalbumin KO mice. This resulted in robust expression of parvalbumin protein in the liver and secretion to the serum (Fig. 2e). One week following the injection, the increase in circulating parvalbumin produced a remarkable decrease of M2 macrophages in Ad-parvalbumin rescued KO mice (Fig. 2f–h). Meanwhile, deficiency of parvalbumin in BMDM did not affect BMDMs polarization to M2 macrophage and parvalbumin treatment inhibited M2 macrophage marker genes expression at similar levels in BMDM from parvalbumin KO mice and WT littermate, which ruled out the possibility that macrophage-endogenous parvalbumin may influence the BMDMs polarization (Fig. 2i).

### Parvalbumin functions as a CSF1R antagonist to inhibit M2 macrophages

**Fig. 3:**
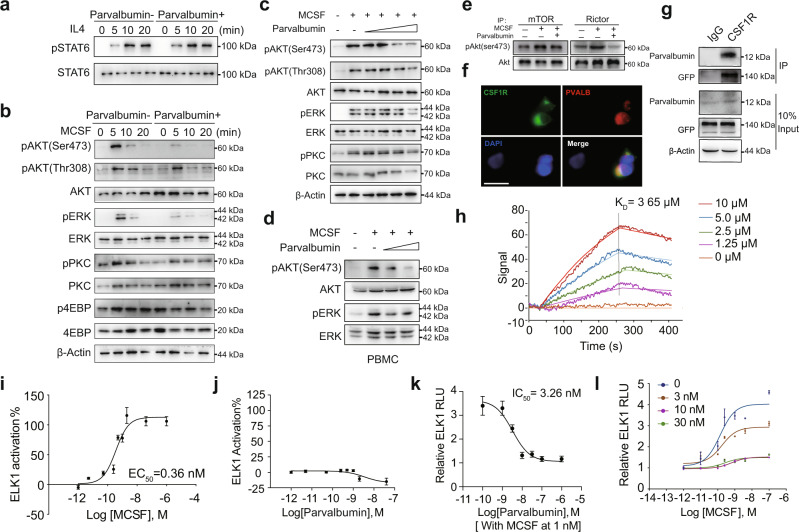
Parvalbumin functions as a CSF1R antagonist to inhibit M2 macrophages. **a** Representative immunoblots showing effects of parvalbumin (100 ng/ml) treatment on IL4-induced STAT6 phosphorylation at indicated time in BMDMs. **b** Representative immunoblots showing effects of parvalbumin (100 ng/ml) treatment on MCSF-induced mTORC2 downstream targets and ERK activation at indicated time in BMDMs. Representative immunoblots showing effects of varying concentrations of parvalbumin treatment on MCSF-induced mTORC2 downstream targets and ERK activation in BMDMs (concentrations of parvalbumin: 10 ng/ml, 50 ng/ml, 100 ng/ml, 200 ng/ml) (**c**) and human PBMCs (concentrations of parvalbumin:10 ng/ml, 100 ng/ml) (**d**). **e** examination of mTORC2 activity through in vitro kinase assay. **f** Co-localization of parvalbumin with transiently transfected CSF1R in COS7 cells. Scale bar, 20 μm. **g** Interaction between parvalbumin and immunoprecipitated GFP-tagged CSF1R. **h** Measurement of the binding affinity between parvalbumin and CSF1R proteins by SPR method. **i** Concentration-response curve of MCSF on CSF1R activation detected by ELK1-Luc in cells stably expressing CSF1R (MCSF EC50 = 0.36 ± 0.03 nM, *n* = 3 per group). **j** Concentration-response curve of parvalbumin on ELK1-Luc, showing that parvalbumin alone does not activate CSF1R (*n* = 3 per group). **k** Concentration-response curve of parvalbumin on CSF1R activation in the presence of MCSF (parvalbumin IC50 = 3.26 ± 0.06 nM) (*n* = 3 per group). **l** Concentration-response curve of parvalbumin on CSF1R activation in the presence of increasing concentrations of parvalbumin, showing that parvalbumin is a noncompetitive antagonist of CSF1R (*n* = 3 per group). Data are represented as mean ± SEM. Source data are provided as a Source Data file.

M2 macrophages are maintained mainly by IL4-STAT6 and MCSF-mTORC2 signaling^9,10^. To assess the mechanism of parvalbumin on M2 macrophage regulation, we first examined the influence of parvalbumin on these two pathways in BMDMs. Surprisingly, parvalbumin (100 ng/ml) did not affect IL4-induced STAT6 phosphorylation (Fig. 3a), while it greatly abrogated MCSF-induced mTOCR2 activation, as shown by reduced phosphorylation of mTORC2 downstream targets AKT (Ser473) and PKC(Ser657) but not mTORC1 targets 4EBP (Thr37/46) in a time and dose-dependent manner in both BMDMs and PBMCs (Fig. 3b–d). Furthermore, we performed in vitro kinase assay to directly test the influence of parvalbumin on mTORC2 activity. MCSF-induced phosphorylation of purified AKT at Ser473 by mTORC2 was reduced by parvalbumin (Fig. 3e). At the meantime, MCSF-induced ERK (Thr202/Tyr204) phosphorylation was also inhibited by parvalbumin treatment in both BMDMs and PBMCs (Fig. 3c, d), indicating that parvalbumin may target the MCSF receptor (CSF1R) to influence its downstream signaling. To test this possibility, we transfected COS-7 cells with CSF1R-GFP and then evaluated parvalbumin binding to these cells using fluorescent-labeled parvalbumin. We found that parvalbumin bound to CSF1R-expressing cells, but not non-expressing cells, indicating specific binding of parvalbumin with CSF1R (Fig. 3f). Indeed, by pull-down experiments, the interaction between exogenously expressed CSF1R and parvalbumin was confirmed in HEK293T cells (Fig. 3g). To further measure the binding affinity between parvalbumin and CSF1R, we employed a Surface Plasmon Resonance (SPR) assay and obtained a KD value of 3.65 μM for these two proteins interaction (Fig. 3h). Based on these findings, we hypothesized that parvalbumin might function as an endogenous antagonist of CSF1R. Supporting this notion, MCSF activated CSF1R with an EC50 of 0.36 nM as assayed by ERK downstream ELK1 reporter assay, similar to the previous report^29^ (Fig. 3i). parvalbumin per se did not impact signaling through CSF1R either positively or negatively, indicating that it is not an inverse agonist (Fig. 3j). However, parvalbumin fully inhibited MCSF-induced CSF1R activation with an IC50 of 3.26 nM (Fig. 3k). Furthermore, parvalbumin reduced the magnitude of maximal CSF1R activation by MCSF and this inhibition could not be overcome by increasing concentrations of MCSF (Fig. 3l). These data clearly showed that parvalbumin functions as a non-competitive antagonist of CSF1R.

### Parvalbumin blocker which blocks Parvalbumin and CSF1R interaction, promotes M2 macrophage polarization

**Fig. 4:**
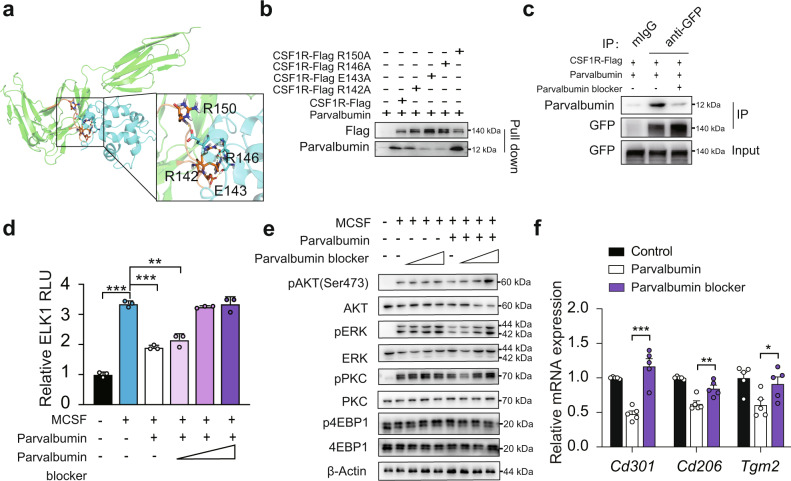
Parvalbumin blocker which blocks Parvalbumin and CSF1R interaction, promotes M2 macrophage polarization. **a** Proposed docking interface between parvalbumin and CSF1R based on molecular docking. CSF1R and parvalbumin are shown in green and blue respectively. The fragment M141-Y154 of CSFR1 is highlighted in orange. The residues in hydrogen bond network are shown in sticks. **b** CSF1R E143A and R146A abrogated the interaction with parvalbumin. **c** Parvalbumin blocker (1 μg/ml) blocked parvalbumin-CSF1R interaction. **d** Effect of parvalbumin blocker on ELK1 luciferase reporter activity in 293 T transfected with CSF1R (concentrations of parvalbumin blocker: 0.1 μg/ml, 1 μg/ml, 10 μg/ml, *n* = 3 per group). Effect of M-CSF with or without parvalbumin was shown (*n* = 3 per group). *P* value: <0.0001, <0.0001 and 0.0009. Parvalbumin blocker rescued parvalbumin suppression of M2 macrophage activation. MCSF-induced mTORC2 downstream targets and ERK activation (concentrations of parvalbumin blocker: 0.1 μg/ml, 1 μg/ml, 10 μg/ml) (**e**) and M2 macrophage marker gene expression (concentrations of parvalbumin blocker: 1 μg/ml) (**f**) (*n* = 5 per group) in BMDM were shown. *P* value: 0.0003, 0.0100, 0.0415. Data are represented as mean ± SEM. *P* values were determined by unpaired two-tailed Student’s *t* test (**d** and **f**). *p < 0.05, **p < 0.01, ***p < 0.001. Source data are provided as a Source Data file.

We then adopted the molecular docking method to map the surface regions of parvalbumin-CSF1R interaction and found that R142, E143, R146 and R150 in M141-Y154 region of CSF1R seem to be necessary for this interaction (Fig. 4a). Indeed, CSF1R-R143A and R146A mutation abrogated the interaction of parvalbumin with CSF1R in pull-down assay (Fig. 4b). The M141-Y154 region of CSF1R (MREGGRQVLRKTVY) was then synthesized as a recombinant pepetide and was used as a parvalbumin blocker to antagonize parvalbumin-CSF1R interaction. Indeed, when parvalbumin blocker was added, the interaction between exogenously expressed CSF1R and parvalbumin was dramatically blocked in HEK293T cells (Fig. 4c). Consistently, parvalbumin suppression of MCSF-induced mTORC2 and ERK activation was also neutralized by parvalbumin blocker as shown by ELK1 reporter (Fig. 4d) and phosphorylation of AKT (Ser473), PKC (Ser657) and ERK (Thr202/Tyr204) in BMDMs (Fig. 4e). As a result, markers of M2 macrophages in polarized BMDM were substantially rescued from parvalbumin suppression as determined by real-time PCR (Fig. 4f).

### Parvalbumin suppresses thermogenesis through M2 macrophages

**Fig. 5:**
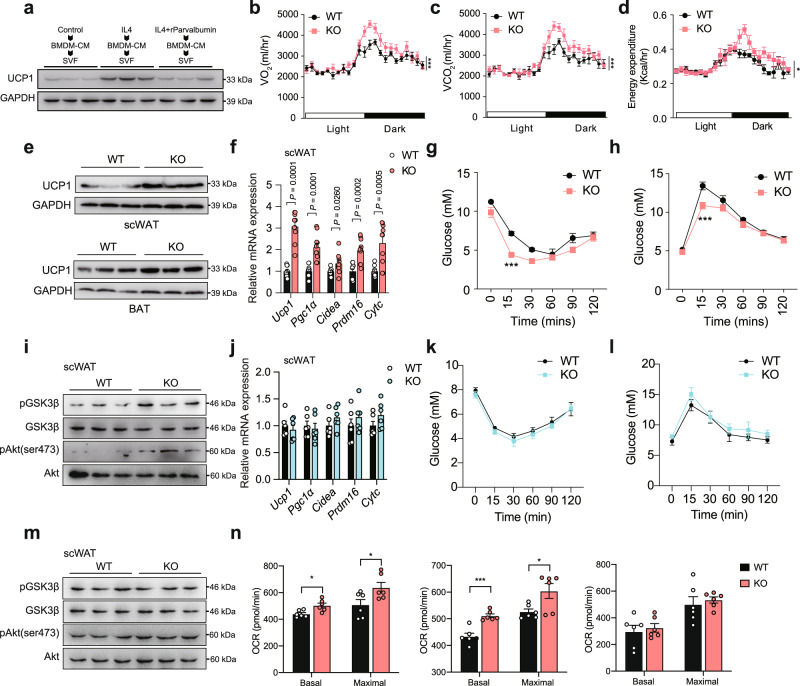
Effects of parvalbumin KO on thermogenesis, insulin sensitivity, and glucose homeostasis. **a** Immunoblots of UCP1 in primary adipocytes after exposure to CM for 24 h (*n* = 3; representative of 3 biological replicates per group). Oxygen consumption (VO_2_) (**b**), carbon dioxide production (VCO_2_) (**c**), and energy expenditure (**d**) of WT and male parvalbumin KO mice (*n* = 6 per group). **e** UCP1 protein expression in scWAT and BAT of WT and parvalbumin-KO mice under room temperature. **f** Representative thermogenic gene expression in scWAT of WT and parvalbumin KO mice (*n* = 10 per group) under room temperature. Insulin tolerance (**g**) and glucose tolerance (**h**) test of WT and parvalbumin KO mice under room temperature (*n* = 8 per group). **i** Representative immunoblots showing effects of parvalbumin KO on insulin signaling in scWAT under room temperature. **j** Representative thermogenic gene expression in scWAT of male WT and parvalbumin KO mice (*n* = 6 per group) under thermoneutrality condition. Insulin tolerance (**k**) and glucose tolerance (**l**) test of male WT and parvalbumin KO mice under thermoneutrality condition (*n* = 6 per group). **m** Representative immunoblots showing effects of parvalbumin KO on insulin signaling in scWAT under room temperature. **n** Basal and maximal OCR of BAT, scWAT, and muscle tissues from parvalbumin KO and WT mice were determined by Seahorse (*n* = 6 per group). *P* value: 0.0106, 0.0385, 0.0003, 0.0258. Data are represented as mean ± SEM. *P* values were determined by unpaired two-tailed Student’s *t* test (**f**, **j**, and **n**), two-way ANOVA with Tukey’s post hoc tests (**g**, **h**, **k**, and **l**), and ANCOVA using weight as a covariable (**b**–**d**). *p < 0.05, **p < 0.01, ***p < 0.001. Source data are provided as a Source Data file.

M2 macrophages have been shown to promote brown and beige adipose tissues thermogenesis and improve systematic insulin sensitivity^30,31^. Our results showed that parvalbumin suppressed M2 macrophage polarization that affected BAT and scWAT thermogensis, which prompt us to investigate whether parvalbumin played a role in this setting. We first used cell systems to investigate whether co-culture of parvalbumin-treated macrophage with adipocytes could affect thermogenic genes expression. BMDMs were treated with IL4 or IL4+ parvalbumin for 24 h, and the conditioned medium (CM) was collected and applied to primary adipocytes differentiated from SVF of scWAT. UCP1 protein levels were lower in adipocytes treated with CM from IL4+ parvalbumin group than with CM from IL4 group (Fig. 5a). Meanwhile, when rPVLAB was applied directly to primary adipocytes, there was no detectable effect of parvalbumin on the regulation of thermogenic genes such as *Ucp1*, *Pgc1α* and *Cidea* at varying doses tested (Fig. s3). We next employed the parvalbumin KO mice to further explore parvalbumin’s function on thermogenesis in vivo. Indeed, whole body indirect calorimetry analyses revealed significantly augmented oxygen consumption (VO_2_), carbon dioxide production (VCO_2_) and energy expenditure (Fig. 5b–d) in parvalbumin-KO mice compared with WT littermate, indicating increased energy expenditure. Protein level of UCP1 was increased in BAT and scWAT (Fig. 5e). Real-time PCR also confirmed the increased expression of important thermogenic genes such as *Ucp1*, *Pgc1α, Cidea, Prdm16 and Cytc* in scWAT and BAT (Figs. 5f and s4a). As a result, body weight was slightly decreased in parvalbumin-KO mice comparing to WT mice under both regular diet (RD) and high-fat-diet (HFD) conditions (Fig. s4b, c), while movement and food intake were unchanged in these mice (Fig. s4d, e).

M2 macrophage in adipose tissue not only promotes thermogenesis but also increases insulin signaling to improve systemic insulin sensitivity. We performed glucose tolerance test and analyzed insulin signaling in muscle, liver, scWAT and BAT in WT and parvalbumin KO mice. parvalbumin KO mice presented improved insulin sensitivity/glucose homeostasis and increased insulin signaling as shown by the phosphorylation of AKT and GSK3 (Figs. 5g–i and Fig. s4f). However, when mice were housed at thermoneutral condition (30 °C), the increase in insulin sensitivity/glucose homeostasis and insulin signaling was largely compromised (Fig. 5j–m, Fig. s4g, h). These results suggested that although parvalbumin may directly influence insulin signaling, its effect relied largely on the regulation of thermogenesis. We also used tissue ex vivo respiration measurement with seahorse to directly test the oxygen consumption rate (OCR) in scWAT, BAT and muscle. the basal and maximum oxygen consumption were higher in BAT and scWAT but not in muscle of parvalbumin-KO mice than WT mice, suggesting increased scWAT and BAT thermogenesis (Fig. 5n). These results demonstrate that parvalbumin deficiency promotes energy expenditure and prevents mice from insulin resistance.

**Fig. 6:**
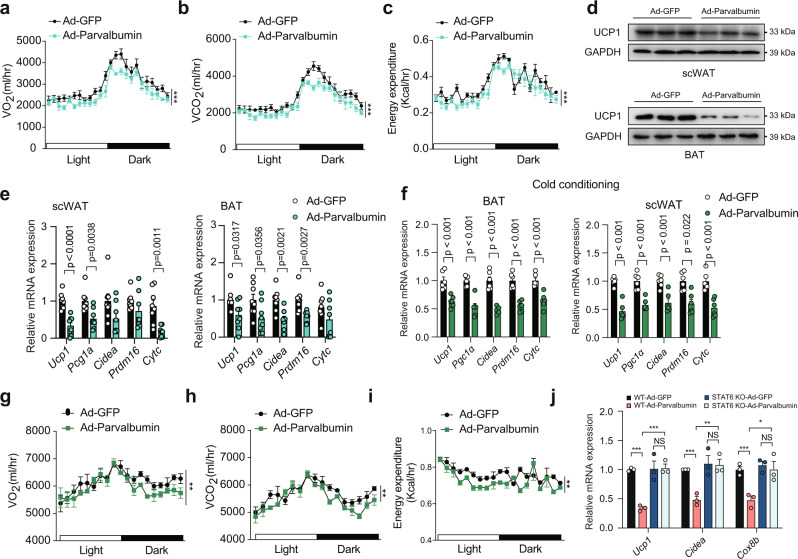
Parvalbumin suppresses thermogenesis through M2 macrophages in mice. Male parvalbumin KO mice were intravenously injected with Ad-parvalbumin or Ad-GFP through the tail vein. VO_2_ (**a**), VCO_2_ (**b**), energy expenditure (**c**), UCP1 protein expression (**d**) and representative thermogenic gene expression (**e**) in scWAT and BAT were examined (*n* = 8 per group). Male parvalbumin KO mice were intravenously injected with Ad-parvalbumin or Ad-GFP through the tail vein and fed under cold conditions. representative thermogenic gene expression (**f**) in scWAT and BAT, VO_2_ (**g**), VCO_2_ (**h**), and energy expenditure (**i**) were examined (*n* = 8 per group). **j** Representative thermogenic gene expression in WT and STAT6 KO mice injected with Ad-GFP or Ad-parvalbumin (*n* = 3 per group). *P* value: <0.0001, 0.0008, 0.0042, 0.0006, 0.0059, 0.0266. Data are represented as mean ± SEM. *P* values were determined by unpaired two-tailed Student’s *t* test (**e**, **f**, and **j**) and ANCOVA using weight as a covariable (**a**–**c** and **g**–**i**). *p < 0.05, **p < 0.01, ***p < 0.001. Source data are provided as a Source Data file.

On the contrary, the increase in circulating parvalbumin by Ad-parvalbumin injection produced a remarkable decrease in energy expenditure (Fig. 6a–c), thermogenic genes expression and UCP1 protein expression in parvalbumin KO mice (Fig. 6d, e), suggesting that circulating parvalbumin rescued the parvalbumin deficient phenotype and was critical for thermogenic regulation. To test whether parvalbumin could affect adaptive thermogenesis under cold exposure, we injected mice with Ad-GFP and Ad-parvalbumin and subjected mice to cold (4 °C) exposure to boost thermogenesis. We found that VO_2_, VCO_2_, energy expenditure, and thermogenic genes expression were suppressed in Ad-parvalbumin-injected mice compared with Ad-GFP-injected mice under cold exposure (Fig. 6f–i).

Next, we used STAT6-KO mice, a specific M2 macrophage defect model, to directly investigate the requirement of M2 macrophage in parvalbumin-induced thermogenic suppression. Loss of STAT6 abrogated the effect of parvalbumin on thermogenic genes expression in scWAT (Fig. 6j). Furthermore, thermogenic genes expression was induced at similar levels by CL316243 treatment in primary adipocytes of parvalbumin KO mice and WT littermate, which ruled out the possibility that scWAT-endogenous parvalbumin may influence the beige adipocytes function (Fig. s5). These observations suggested that parvalbumin inhibits expression of thermogenic genes through M2 macrophage.

### Serum level of parvalbumin correlates with mouse and human obesity

**Fig. 7:**
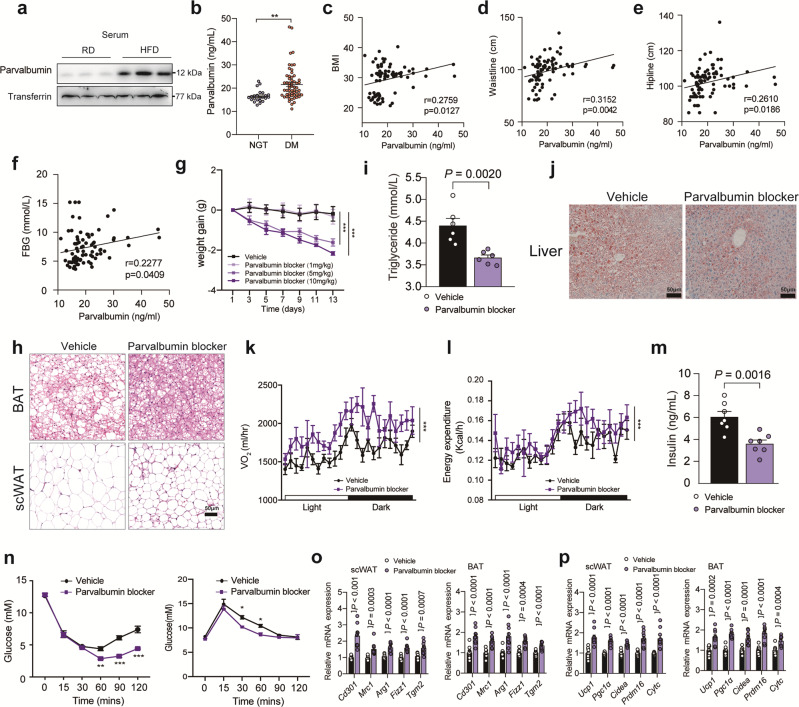
Parvalbumin blocker ameliorates HFD-induced obesity. **a** Parvalbumin protein levels in serum from RD- or HFD-fed mice. **b** Serum parvalbumin levels in lean and obese individuals (lean, *n* = 23; obese, *n* = 58). *P* value: 0.0011. The correlation between BMI (**c**), waistline (**d**), hipline (**e**) and FBG (**f**) with serum parvalbumin. HFD-fed mice were injected daily with vehicle (*n* = 14) or parvalbumin blocker (5 mg kg-1, *n* = 13) for 2 weeks. The weight gain (**g**), representative images of H&E staining of BAT and scWAT (**h**), circulating triglycerides (**i**), representative images of oil O Red staining of liver (**j**), VO_2_ (**k**), energy expenditure (**l**), and circulating insulin were determined (*n* = 6 per group). scale bar = 50 μm. **n** Insulin tolerance test and glucose tolerance test of vehicle or parvalbumin blocker injected mice (*n* = 6 per group). *P* value: 0.0036, <0.0001, <0.0001, 0.0215, 0.0376. Representative thermogenic (**o**) and M2 macrophage marker (**p**) gene expression in scWAT and BAT of vehicle or parvalbumin blocker injected mice (*n* = 10 per group). Data are represented as mean ± SEM. *P* values were determined by unpaired two-tailed Student’s *t* test (**b**, **o**, and **p**), two-way ANOVA with Tukey’s post hoc tests (**g** and **n**), ANCOVA using weight as a covariable (**k** and **l**) or Pearson correlation analysis (**c**–**f**). *p < 0.05, ***p < 0.001. Source data are provided as a Source Data file.

Obesity is characterized by decreased M2 macrophages in adipose tissue, which leads to insulin resistance^32,33^. We next explored whether serum level of parvalbumin correlates with mouse and human obesity. Indeed, serum levels of parvalbumin were dramatically elevated in HFD-fed mice compared with RD-fed mice (Fig. 7a), indicating its correlation with obesity. Remarkably, we found that serum levels of parvalbumin in obese human individuals were significantly higher than that of non-obese individuals (Fig. 7b). A positive correlation between serum levels of parvalbumin and BMI, waistline, hipline and fasting blood glucose (FBG) was also observed (Fig. 7c–f). Thus, our data indicate that serum level of parvalbumin correlates with both mouse and human obesity.

### Parvalbumin blocker ameliorates HFD-induced obesity

Accumulated evidence suggests that activation of brown and beige adipose tissue thermogenesis ameliorates obesity and related insulin resistance. Our data indicate that parvalbumin blocker might represent a valuable therapeutic approach for anti-obesity and prompt us to evaluate its effect on obesity. We delivered parvalbumin blocker (1, 5 and 10 mg/kg/day) through intraperitoneal (i.p.) injection to HFD-fed mice for 14 days. A significant reduction of body weight gain was observed in parvalbumin blocker-injected mice (5 and 10 mg/kg/day) versus scramble-injected mice (Fig. 7g). Meanwhile, adiposity in BAT and scWAT, hepatic steatosis as well as plasma triglycerides (TAG) levels were also lower in parvalbumin blocker treated mice (Fig. 7h–j). Whole body indirect calorimetry analyses revealed augmented VO_2_, and energy expenditure (Fig. 7k, l) in parvalbumin blocker-injected mice (5 mg/kg/day) versus scramble-injected mice, indicating increased energy expenditure, while food intake and activity were not changed (Fig. s6a, b). When mice were housed at a thermoneutral environment (30 °C), parvalbumin blocker injection failed to reduce the body weight of mice, indicating that parvalbumin blocker reduced weight gain through thermogenesis (Fig. s6c). Furthermore, treatment with parvalbumin blocker significantly lowered insulin levels and ameliorated insulin resistance in HFD-fed mice (Fig. 7m, n). Accordingly, we found that expression of M2 macrophage marker genes as well as thermogenic genes in scWAT and BAT was significantly elevated in parvalbumin blocker treated mice (Fig. 7o, p). Importantly, the beneficial effect of parvalbumin blocker was parvalbumin dependent as parvalbumin deficiency abolished these effects (Fig. s7). These results demonstrated that parvalbumin blocker might have potential values in the treatment of obesity, which will need further evaluation in humans.

## Discussion

Accumulating evidence has revealed that regulators of M2 macrophage activation could influence thermogenesis, although the mechanism is still under debate. For example, cold-induced adiponectin, FGF21 and CXCL14 promote M2 macrophage activation and glucose/lipid homeostasis in obese mice^11,12,34^. Interestingly, exercise-induced Metrnl also increases M2 macrophages and promotes beige thermogenesis^16^. Thus, targeting M2 macrophages may represent a promising strategy to ameliorate obesity. We described a new circulating factor parvalbumin, which suppresses thermogenesis through M2 macrophage. parvalbumin functions as a non-competitive antagonist of CSF1R to inhibit MCSF-induced mTORC2 activation and M2 macrophage activation. Most Importantly, blocker that blocks parvalbumin and CSF1R interaction not only rescued M2 macrophage activation, insulin sensitivity and thermogenesis but also ameliorated obesity in mice.

Parvalbumin is a classical member of the EF-hand protein superfamily and is expressed in various neurons such as GABAergic interneurons (reticular thalamus)^35^, chandelier and basket cells (cortex)^36^, Purkinje cells and molecular layer interneurons (cerebellum)^37^, and pyramidal cells (hippocampus)^38^. PVLAB interneurons are critical for emergent gamma-frequency rhythmicity and are correlated with schizophrenia and Alzheimer’s disease^39–41^. Meanwhile, GAD67 downregulation in parvalbumin-positive neurons also affects food seeking and motivation in mice, indicating its potential effect on food intake and activity^42,43^. However, whether parvalbumin itself plays a role in food intake and activity is still elusive. Our results clearly show that whole body parvalbumin knockout does not affect food intake and motor activity. While whole body parvalbumin deficiency may lead to adaptive/homeostatic changes (e.g. altered spine morphology, changes in mitochondria content) in the neurons^44^, which may account for the lack of influence on food intake and activity, the detailed mechanism will need to employ brain region specific KO mice to clarify and is out of the scope of current investigation.

In addition to neurons, parvalbumin is highly expressed in fast-twitch muscle fibers and much less expressed in kidney, parathyroid and BAT. It is reported that contraction inhibits muscle expression of parvalbumin, which is involved in relaxation of fast-twitch muscle fibers^45,46^. Parvalbumin is also expressed in the early part of the distal convoluted tubule in the kidney and plays a direct role in the trans-epithelial handling of Ca^2+^ and Mg^2+^^47^. Although mainly expressed in muscle in mice, parvalbumin is highly enriched in cerebellum and kidney in humans (http://biogps.org). As a result, the circulating parvalbumin in humans may come from the contribution of multiple tissues and could not be identified as a muscle-derived factor in humans. At the meantime, data of parvalbumin expression levels in human muscle between sedentary and exercise group from public gene expression database are controversial. Some reported limited changes of parvalbumin expression levels in human muscle^48,49^, while other such as data from GSE 9103 showed downregulated parvalbumin expression in exercised individuals (Fig. s2d). Although we reported an upregulation of circulating levels of parvalbumin, which correlated with human obesity, we could not conclude that the upregulation of parvalbumin in the serum was mediated by muscle secretion. Despite the difference in parvalbumin expression in mice and humans, we were able to prove that parvalbumin suppressed M2 macrophage polarization in both mice and humans, thus targeting parvalbumin may serve as a strategy to combat against obesity in humans, which will need further evaluation.

It has been reported that parvalbumin could be secreted into the blood stream^50^, its function as a circulating factor is still unknown. Here, we found that serum parvalbumin is decreased in exercised mice compared to sedentary mice, which correlates with decreased expression of parvalbumin in muscle but not the kidney. Take consideration of the enriched expression of parvalbumin in muscle compared to kidney and other peripheral tissues, it is possible that circulating parvalbumin is secreted from muscle cells. Indeed, we verified that parvalbumin can be secreted into mediums from primary muscle fibers or muscle tissues through small EVs, further suggesting that muscle is an important source of circulating parvalbumin in mice. Although we could not fully rule out the possibility that other tissue such as the kidney and parathyroid gland may also contribute to the circulating parvalbumin, we focused on the function of circulating parvalbumin by showing that parvalbumin knockout increases thermogenesis and insulin sensitivity, which is blunted by increasing parvalbumin in serum of KO mice.

MCSF synergizes with IL-4 to induce M2 activation by promoting mTORC2 signaling^51^, which integrates systemic metabolic and thermogenic responses^30,52^. Besides, MCSF is also critical for macrophage survival which depends on PI3K and AKT^53^. Our data showed that parvalbumin inhibits M2 activation without having any effects on macrophage proliferation and survival. One possible explanation could be that parvalbumin serves as a non-competitive antagonist of MCSF to specifically suppress mTORC2-AKT pSer473 cascade while the PI3K-AKT pThr308 cascade is not influenced. Meanwhile, in the absence of MCSF signaling, elevated granulocyte macrophage colony-stimulating factor (GMCSF) expression and as a result, persistent activation of STAT5 could compensate for the lack of MCSF and induces M1 macrophage activation^52,54^. Indeed, we also observed that M1 macrophage activation is greatly boosted by parvalbumin treatment. Whether parvalbumin could promote GMCSF signaling to compensate for MCSF inhibition will be of great interest and need further investigation.

Muscle has emerged as an important secretory organ recently. Besides myokines secreted into circulation from muscle in free forms, several secretory factors are also inserted into small vesicles for secretion. Muscle-derived small extracellular vesicles (small EVs) contain microRNA (miRNA) such as miR-1, miR-206, and miR-133 which contribute to muscle cell development and insulin secretion in pancreatic islets^55–57^. Furthermore, protein components such as CD9, CD81, CD44, and myoferlin are also found in small EVs secreted from muscle and play key roles in muscle cell development^58,59^. Most importantly, the component of muscle-derived small EVs varies depending on different physiological or pathological conditions. A high-fat diet feeding accelerates the release of small EVs from muscle tissues in mice which induces myoblast proliferation^60^. Acute exercise also stimulates muscle-enriched miR-133 and miR-1 secretion through small EVs^61^. Furthermore, Exercise induces secretion of small EVs-contained proteins Destrin, eukaryotic initiation factor 4A1, Tetraspanin CD151, and Integrin beta 5 (ITGB5) from human muscles and might target the liver and other tissues to regulate their functions^62^. Thus, small EVs provide a novel mechanism for muscle-derived factor secretion and tissue crosstalk during exercise. Although parvalbumin was not identified in these reports probably due to the limitations in mass spectrometry technology, our finding that parvalbumin functions as a new muscle small EVs-mediated secretory protein to regulate macrophage polarization in adipose tissue further supports this notion. The interaction of small EVs with different cell types is a well-observed phenomenon but the process and mechanism directing small EVs interaction with recipient cells remain poorly understood^63^. This process may involve direct association of small EVs with receptors at the plasma membrane without gaining entry or internalization by endocytosis or membrane fusion^63,64^. Although the detailed mechanism of how small EVs-secreted parvalbumin interacts with macrophage target is still unknown, a possible explanation might shed light on the understanding of this process. Sialic acids enriched on small EVs^65^ may be recognized by macrophage-expressed CD169^66^ which facilitates the binding of muscle-derived small EVs to macrophages. Then, parvalbumin released from these captured small EVs could interact with CSF1R to regulate macrophage polarization. The targeting of small EVs-derived parvalbumin on macrophage is an interesting topic that will need further evaluation.

## Methods

### Clinical samples

Two cohorts including 23 lean persons (age 38 ± 8 years, BMI 23.4 ± 1.7 kg/m^2^) and 58 obese patients (age 42 ± 10 years, BMI 32.0 ± 3.1 kg/m^2^) were investigated (clinical characteristics in Supplementary Table 1). Serum samples were collected from the Department of Endocrinology, Shanghai General Hospital, and used for ELISA analysis for parvalbumin levels. All studies were approved by the regional board of ethics of Shanghai General Hospital, Shanghai Jiao Tong University (Permit Number: 2017KY209), and informed written consent was obtained from each participant.

### Animals

All animal studies were approved by the Animal Experiment Committee of Tongji University and in accordance with the guidelines of the School of medicine, Tongji University (Permit Number: SHDSYY-2019-TJ15028). This study was carried out in strict accordance with the recommendations in the Guide for the Care and Use of Laboratory Animals of the Ministry of Science and Technology of the People’s Republic of China. C57BL/6 N mice were purchased from Slack Laboratory Animal Co., Ltd. (SLAC, Shanghai, China). Parvalbumin-KO mice on the C57BL/6 N background were purchased from Cyagen Biosciences (Serial Number: KOCMP-21108-Pvalb). Briefly, parvalbumin KO mice was generated using the CRISPR/Cas9 system, which was validated by Sanger sequencing in F0 generation. The gRNA1 (TATGTATGCCCGACAAGTACAGG) and gRNA2 (TGTACAGGGGGATCGATCAGTGG) targeting exon 3 of the parvalbumin gene was electroporated into fertilized zygotes together with the Cas9 protein. Reverse-transcription polymerase chain reaction and Western blot analysis approved the knockout of parvalbumin in these mice. STAT6-KO mice on the BALB/c background (Jackson Laboratory, strain #:002828) were kindly provided by Prof. Rui He from Fudan University, Shanghai, China. Littermate wildtype mice were used as controls.

Experimental/control animals mice aged 8–16 weeks were bred seperately in ventilated cages in a temperature-controlled facility (22 ± 2 °C) with a 12 h (h) light/12 h dark cycle (lights on 6:00–18:00), relative humidity (45–65%), and free access to food and water under specific pathogen-free (SPF) conditions.

For intramuscular (im) viral injections, mice were anesthetized with 20 mg/ml Avertin (10 μl/g), and 50 μl of viral preparations with titers from 10 × 10^8^ IU/ml was injected into 4 to 6 sites in the soleus muscles. All animals were euthanatized for analysis 7 days after viral administration.

For exercise model construction, C57BL/6 N mice were subjected to running on a motorized, speed-controlled treadmill system (Columbus Instruments, Columbus, OH) for 36 days (12 m/min, 1 h/day for 6 days/week) and the running distance is about 720 m for 1 h^67^. The inclination angle was 10%. For metabolic studies, 8 weeks old male parvalbumin KO or littermate WT mice were fed on a RD (5% fat; Research Diet, D12450, New Brunswick, NJ, USA) or a HFD (60% fat, D12492, Research Diets) at ambient temperature.

For glucose tolerance test (GTT), mice were fasted for 12 h (from 20:00 to 08:00) and injected intraperitoneally with glucose (1.5 g/kg for HFD-fed mice and 1 g/kg for RD-fed mice). For insulin tolerance test (ITT) experiments, mice were fasted for 3 h (from 10:00 to 13:00) and injected intraperitoneally with insulin (1 U/kg for HFD-fed mice and 0.75U/kg for RD-fed mice). Blood from anesthetized mice (4% isofluorane at euthanization) was collected by cardiac puncture and blood from alive mice was collected from a tail snip. Serum was collected from the blood which was stored at room temperature for 2 h and centrifuged at 1000 g for 15 min. For adenovirus injection, 1 × 10^9^ plaque forming unit (pfu) Ad-parvalbumin or Ad-GFP were delivered to 8-week-old male parvalbumin-KO and WT mice by tail vein injection. Mice were injected with adenovirus on day 0 and euthanatized on day 8. The metabolic parameters of mice injected with adenovirus were measured 7 days after injection^68,69^.

For blocker injection, parvalbumin-KO or WT mice fed on a HFD-diet were intraperitoneally injected with the indicated concentration of parvalbumin blocker (1, 5, or 10 mg/kg, per day, for 2 weeks).

### Chemicals and antibodies

All chemicals were obtained from Sigma-Aldrich unless otherwise specified. The following antibodies were used in this study: rabbit anti-UCP1 (Abcam, ab155117, GR3233606-10 1:2000), anti-Parvalbumin (ABclonal, A13538, Lot0054370201 1:1000), anti-Transferrin (Abbkine, ABM40235, 1:1000), anti-pSTST6 (Cell Signaling Tech, 56554, 1:2000), anti-STAT6 (Cell Signaling Tech, 9362, 1:2000), anti-pAKT (Ser473) (Cell Signaling Tech, 4060, 1:2000), anti-pAKT (Thr308) (Cell Signaling Tech, 13038,1:2000), anti-AKT (Cell Signaling Tech, 2920, 1:2000), anti-GAPDH (Santa Cruz, sc32233, 1:1000), anti-GFP (Proteintech, 50430-2-AP, 1:1000), anti-pERK (Cell Signaling Tech, 4370, 1:2000), anti-ERK (Cell Signaling Tech, 4695, 1:2000), anti-pPKC (Abcam, ab180848, 1:2000), anti-PKC (Abcam, ab179522, 1:2000), anti-p4EBP1 (Cell Signaling Tech, 2855, 1:2000), anti-4EBP1 (Cell Signaling Tech, 9452, 1:2000), anti-ACTB (Sigma Aldrich, A3854, 1:10000), anti-Flag (Sigma-Aldrich, F7425, 1:10000), HRP-conjugated goat anti-Rabbit IgG (Cell Signaling Tech, 7074, 1:10000), anti-pGSK-3β (Cell Signaling Tech, 5558, 1:2000), anti-GSK-3β (Cell Signaling Tech, 12456, 1:2000). Rictor antibody (Abcam, ab70374, 1:100), mTOR antibody (Cell Signaling Tech, 2983, 1:100) (Supplementary Table 2).

### Cell culture

Primary beige adipocytes were induced by treating confluent stromal vascular fraction (SVF) of scWAT with 0.5 mM isobutylmethylxanthine, 125 nM indomethacin, 2 μg/ml dexamethasone, 850 nM insulin, 1 nM T3 and 0.5 μM rosiglitazone. Two days after induction, cells were switched to maintenance medium containing 10% FBS, 850 nM insulin, 1 nM T3 and 0.5 μM rosiglitazone for another 4 days. HEK293A, HEK293T, and COS-7 cells were obtained from the American Type Culture Collection (ATCC) and maintained in Dulbecco’s Modified Eagle Medium (DMEM) containing 10% fetal bovine serum (FBS) and penicillin/streptomycin (PS) (100 U/ml and 100 μg/ml). No commonly misidentified cell line was used in this study. All cell lines were routinely tested negative for mycoplasma contamination. Bone marrow was isolated and bone-marrow macrophages (BMDMs) were produced after 5–7 days in RPMI 1640 media containing with 10% FBS, 1% penicillin-streptomycin and 25 ng/ml recombinant murine M-CSF (416-ML, R&D systems).

### Seahorse XFe24 measurements

An XF24 Islet Capture Microplate Screen was loaded onto the Screen Applicator Tool and the tool was positioned with the screen facing up. A total of 4 mg of tissue (2 tissue punches) from each fat pad were placed onto the screen with forceps, resulting in three tissue replicates per fat pad. The screen was then snapped into the appropriate well of the Islet Microplate. Rinse medium was added to each well after application of the tissue and screen. After applying all tissue samples and MA Medium, each well was rinsed two additional times. After removal of the final medium, 500 μl Supplement XF DMEM assay medium, with 3 mM glucose and 1% FBS, was added to all sample and control wells.

The mitochondrial stress test utilizes sequential injections of oligomycin (5 μM), Carbonyl cyanide 4-(trifluoromethoxy) phenylhydrazone (1 μM, FCCP), and rotenone/antimycin A in combination (5 μM). The parameters of basal respiration and maximal respiration were automatically calculated by the WAVE software (Agilent). To eliminate the influence caused by mitochondrial-derived CO_2_, we performed seahorse assay with the Seahorse XF Glycolytic Rate Assay kit (101122-100, Agilent Technologies).

### BMDM culture and conditioned medium (CM) preparation

Bone marrow was isolated and bone-marrow macrophages (BMDMs) were produced after 5–7 days in RPMI 1640 media containing with 10% FBS, 1% penicillin-streptomycin and 25 ng/ml recombinant murine M-CSF (416-ML, R&D systems).

For CM collection, BMDM were plated and treated with PBS, IL4, or IL4 combined with parvalbumin for 24 h. Then the medium was changed to FreeStyle expression medium (12338026; Gibco, Thermo Fisher Scientific, USA). The CM were collected 24 h later, centrifuged at 15000 × *g* for 5 min, and filtered through a 0.22-mm filter. Adipocytes were exposed to fresh medium mixed with CM at a ratio of 1:1 (v/v) for 72 h during the last differentiated process.

### Cell viability and caspase-3/7 activity assays

A total of 1 × 10^4^ cells were plated in 96-well plates and treated IL4 with or without parvalbumin for 24 h. Cell viability was assayed by using the CellTiter-Glo® luminescence assay (Promega, Madison, WI) according to the manufacturer’s instructions. Caspase-3/7 activity was measured by the Caspase-Glo 3/7 Assay (Promega) that uses a caspase-3/7 (DEVD) substrate. Measurements were performed by using a luminescence reader (TECAN), according to the recommended protocol by Promega.

### Muscle fiber and muscle tissue conditional medium

For the preparation of muscle fiber conditional medium, extensor digitorum longus (EDL) muscle was isolated from 8-week-old mice, rinsed with DMEM and digested with 0.2% collagenase type 1 for 2 h to isolate single fibers. After 24 h incubation with serum free DMEM, muscle fiber conditional medium was collected and filtered with 0.22 μm filter membrane. For the preparation of the muscle tissue conditional medium, isolated hind leg muscle tissues from 8-week-old mice were rinsed with DMEM and snipped along the direction of skeletal muscle fibers. After rinsing with DMEM and centrifuging at 300 × *g* for 5 min, supernatant was abandoned and serum free DMEM was added for 24 h. Muscle tissue conditional medium was collected and filtered with 0.22 μm filter membrane.

### Human PBMCs

The human PBMCs were obtained from human plasma samples using lymphocyte separation medium (40504ES60, YEASEN, China) according to manufacturer’s instruction and maintained in RPMI 1640 medium with 10% FBS, 1% penicillin/streptomycin/glutamine and 25 ng/ml recombinant human MCSF (216-MC, R&D systems). Human PBMC were cultured in the presence of IL4 or MCSF for 24 h with or without recombinant human parvalbumin (15333-H08E, Sino Biological, China) and subjected to real-time PCR or immunoblot analysis.

### TMT Quantitative proteomics

Sample preparation: The albumin and IgG from 30 μL serum samples were removed using the ProteoExtract Kit according to the protocol. The ultrafiltration was performed using 10 kDa molecular weight cutoff filter to a final volume of 100 μL for each sample depleted of both albumin and IgG. The protein concentration of the supernatant was determined using the BCA protein assay, and then 100 μg protein per condition was transferred into a new tube and adjusted to a final volume of 100 μL with 100 mM TEAB (triethylammonium bicarbonate). 5 μL of the 200 mM DTT was added at 55 °C for 1 h, then 5 μL of the 575 mM iodoacetamide was added to the sample for 1 h in the dark at room temperature.

Protein digestion and TMT labeling: Proteins were tryptically digested with sequence-grade modified trypsin (Promega, Madison, WI), and the resultant peptide mixture was labeled using chemicals from the TMT reagent kit. The labeled samples were combined, desalted using C18 SPE column (Sep-Pak C18, Waters, Milford, MA) and dried in vacuo.

High pH reverse phase separation: The peptide mixture was redissolved in the buffer A (buffer A: 10 mM ammonium formate in water, pH10.0, adjusted with ammonium hydroxide), and then fractionated by high pH separation using an Aquity UPLC system (Waters Corporation, Milford, MA) connected to a reverse phase column (BEH C18 column, 2.1 mm × 150 mm, 1.7 μm, 300 Å, Waters Corporation, Milford, MA). High pH separation was performed using a linear gradient. Starting from 0% B to 45% B in 35 min (B: 10 mM ammonium formate in 90% ACN, pH 10.0, adjusted with ammonium hydroxide). The column flow rate was maintained at 250 μL/min and the column temperature was maintained at 45 °C. Twelve fractions were collected, each fraction was dried in a vacuum concentrator for the next step.

Low pH nano-HPLC-MS/MS analysis: The fractions were resuspended with 32 μl solvent C respectively (C: water with 0.1% formic acid; D: ACN with 0.1% formic acid), separated by nanoLC and analyzed by on-line electrospray tandem mass spectrometry. The experiments were performed on a Nano Aquity UPLC system (Waters Corporation, Milford, MA) connected to a quadrupole-Orbitrap mass spectrometer (Q-Exactive) (Thermo Fisher Scientific, Bremen, Germany) equipped with an online nano-electrospray ion source. 4 μl peptide sample was loaded onto the trap column (Thermo Scientific Acclaim PepMap C18, 100 μm × 2 cm), with a flow of 10 μl/min for 3 min and subsequently separated on the analytical column (Acclaim PepMap C18, 75 μm × 25 cm) with a linear gradient, from 5% D to 30% D in 85 min. The column was re-equilibrated at initial conditions for 5 min. The column flow rate was maintained at 300 nL/min and column temperature was maintained at 45 °C. The electrospray voltage of 2.0 kV versus the inlet of the mass spectrometer was used.

The Orbitrap Fusion mass spectrometer was operated in the data-dependent mode to switch automatically between MS and MS/MS acquisition. Survey full-scan MS spectra (m/z 400–1600) were acquired in Orbitrap with a mass resolution of 60,000 at m/z 200. The AGC taget was set to 500,000, and the maximum injection time was 50 ms. MS/MS acquisition was performed in Orbitrap with 3 s cycle time, the resolution was 15,000 at m/z 200. The intensity threshold was 50,000, and the maximum injection time was 150 ms. The AGC target was set to 150 000, and the isolation window was 2 m/z. Ions with charge states 2+ , 3+, and 4+ were sequentially fragmented by higher energy collisional dissociation (HCD) with a normalized collision energy (NCE) of 37%. In all cases, one microscan was recorded using dynamic exclusion of 30 s. MS/MS fixed first mass was set at 110.

Database Searching: Tandem mass spectra were extracted by Proteome Discoverer software (Thermo Fisher Scientific, version 1.4.0.288). Charge state deconvolution and deisotoping were not performed. All MS/MS samples were analyzed using Mascot (Matrix Science, London, UK; version 2.3). Mascot was set up to search the Uniprot-SwissProt database (Taxonomy: Mus musculus, 16957 entries) assuming the digestion enzyme trypsin. Mascot was searched with a fragment ion mass tolerance of 0.050 Da and a parent ion tolerance of 10.0 PPM. Carbamidomethyl of cysteine and TMT 6plex of lysine and the n-terminus were specified in Mascot as fixed modifications. Oxidation of methionine was specified in Mascot as a variable modification.

Quantitative data analysis: Use the percolator algorithm to control peptide level false discovery rates (FDR) lower than 1%. Only unique peptides were used for protein quantification, and the method of normalization on protein median was used to correct experimental bias, the minimum number of proteins that must be observed to allow was set to 200.

The differentially expressed proteins were illustrated by volcano plot, which was generated using R package ggpubr with log2 FC > 1 or <−1 with *p-value* < 0.01. (Supplementary Data 1).

### Mass spectrometry

To verify the bands identified by anti-parvalbumin in immunoblots (Fig. S1A), the corresponding bands were excised from unstained lanes that were mirror images of the immunoblotted lanes from the same SDS-PAGE gel (from another half of the gel which had not been electroblotted). Gel bands were subjected to in-gel tryptic digestion and samples were analyzed by NanoLC-LTQ Orbitrap. Analysis of the MS2 spectra from both aqueous and gel band-derived parvalbumin standards using Proteome Discover 1.4 (Thermo Fischer) identified two peptides KAIGAF AAADSFDHKK, KVFH ILDKDK corresponding to parvalbumin (Fig. S1D). The mass spectrometry proteomics data have been deposited to the ProteomeXchange Consortium via the PRIDE partner repository with the dataset identifier ProteomeXchange: PXD033582.

### Luciferase reporter assay

HEK293T and COS-7 cells were transfected with ELK1-Luc reporter, RSV-βgal, and indicated plasmids for 24 h. Briefly, cells were lysed using a detergent-containing buffer. Cell debris was removed by microcentrifugation and luciferase activity was measured using a luminometer. Luciferase reporter assay was performed as described^70^.

### Muscle derived small EVs examination

Small EVs release inhibitor GW4869 was dissolved in DMSO at 5 mg/mL and then diluted to 0.3 mg/mL in PBS. Each mouse received 100 μl local injection solution (30 mg/mouse) in both hind leg muscles. Control mice were injected with a 6% DMSO solution in PBS. After 6 h of injection, muscle tissues were cultured in 6 well plate for 12 h. Cell culture medium was collected into a 15 mL centrifuge tube, 3000 × *g*, centrifuged for 10 min. Carefully collect the supernatant and transfer it to a new centrifuge tube and was thoroughly mixed with exosome extraction reagent (Hieff® Quick exosome isolation kit, 41201ES25, Yeason). The centrifuge tube containing the mixture was taken out at 4 °C and centrifuged at 10000 × *g* for 60 min. The supernatant was discarded and precipitation was collected and prepared for immunoblot analysis.

### Nanoparticle tracking analysis (NTA)

We measured the EVs particle size and concentration using nanoparticle tracking analysis (NTA) at VivaCell Shanghai with ZetaView PMX 110 (Particle Metrix, Meerbusch, Germany) and corresponding software ZetaView 8.04.02. Isolated EVs samples were appropriately diluted using 1X PBS buffer (Biological Industries, Israel) to measure the particle size and concentration. NTA measurement was recorded and analyzed at 11 positions. The ZetaView system was calibrated using 110 nm polystyrene particles. Temperature was maintained around 23 °C and 30 °C. Size distribution data were analysed by normalizing the concentration of particles of different diameters with bin widths of 1 nm and then taking the average of each measurement.

### Electron microscopy

Transmission electron microscopy (TEM) was performed to detect the size and morphology of EVs samples. Isolated EVs samples were deposited onto formvar/silicone monoxide coated 200mesh copper grids (Electronmicroscopy Sciences) for 2–3 min, followed by fixation with 3.7% formalin and washed twice with water. The samples were contrasted with 2% Uranyl Acetate (w/v). All the solutions used were 100 kDa filtered to avoid particulate deposits on the grids. The dried grids were viewed using a JEOL 6400 electron microscope.

### Adenoviral amplification and purification

Recombinant Ad-parvalbumin and Ad-GFP adenovirus was generated using the AdEasy system^71,72^. Briefly, linearized shuttle vector containing full-length mouse cDNA for parvalbumin and GFP were transformed into *E. coli* BJ5183AD cells containing the adenoviral backbone plasmid pAdEasy-1 for homologous recombination. Positive recombinants were linearized and transfected into HEK293A cells for virus packaging and propagation. Adenoviruses expressing the candidate gene were purified by Fast Trap Adenovirus Purification and Concentration Kit (EMD Millipore), according to the manufacturer’s instruction.

### Reconstruction of plasmids and preparation of lentivirus

The vector pLV[shRNA]-EGFP-T2A-Puro-U6 was used as a backbone plasmid to reconstruct lentiviral vector containing mouse parvalbumin shRNA#1 CTTGTCTGCTAAAGAAACAAA (VB900058-3078qvu, VectorBuilder, China). The reconstructed plasmid was verified by Sanger sequencing. For lentiviral production, HEK293T cells were co-transfected with control vector or lentiviral plasmid carrying shRNA fragments, along with lentiviral packaging mix (psPAX2 and the pMD2.G plasmid containing VSV-G). The culture medium was collected at 48 and 72 h after transfection, filtered through a 0.45 μm filter, and incubated overnight with polyethylene glycol 8000 (PEG 8000) before being concentrated by centrifugation (4000 × *g* for 20 min at 4 °C; Thermo). Lentivirus was titrated using a titer kit, and virus particles were stored at −80 °C until use. The ratio of lentivirus-infected soleus was observed under an inverted fluorescence microscope (Olympus, Tokyo, Japan).

### Insulin signaling analysis

Mice were fasted overnight for 16 h and injected i.p. with 0.75 U/kg of insulin. 10 min after injection, mice were killed and samples of liver, muscle, scWAT and BAT were snap-frozen in liquid nitrogen and proteins extracted for immunoblot analysis.

### ELISA assay

To measure serum levels of parvalbumin, insulin, and triglycerides, serums were collected from human patients or mice. The serum levels of parvalbumin and insulin were determined by ELISA. The assay kit for human parvalbumin was from Abclonal Technology (RK02169), and mouse insulin was purchased from Crystal Chem (90080). Mouse triglycerides were determined using BioAssay Systems (EGTA006).

### RNA preparationand and real-time PCR

Total RNA (DNA removed with DNA-free DNase Treatment & Removal Reagent) was extracted from tissue or cells using RNA simple Total RNA Kit (Tiangen, Shanghai, China) and reverse transcripted using FastQuant RT kit (Tiangen, Shanghai, China). Real-time PCR was carried out using SuperReal SYBR Green kit (Tiangen, Shanghai, China) and Lightcycler 96 (Roche, Penzberg, Germany). The primer sequences were listed in Supplementary Table 3.

### Pull-down assay and immunoblot analysis

For pull down assay, 106 cells transfected with indicated plasmid were lysed using 400 μl of lysis buffer (Tris-HCL HCl 50 mM, pPH 7.4, Nacl NaCl 150 mM, sodium deoxycholate 0.25%, NP-40 1%, EDTA 1 mM, PMSF 1 mM, Aprotinin 1 mg/ml, leupeptin 1 mg/ml, pepstatin 1 mg/ml) on ice for 30 min and centrifuged at 16113.6 rcf for 15 min. The supernatant was incubated with appropriate primary antibodies overnight and followed by protein A/G plus agarose bead (Santa Cruz Biotech., sc-2003) for another 4 h. After washing three times with ice-cold PBS, parvalbumin (1 ug/ml) was added for 2 h. Agarose beads were washed three times with PBS and followed by SDS-PAGE and immunoblot analysis with indicated antibodies.

Protein lysates from isolated tissues or cultured cells were extracted using RIPA buffer with protease and phosphatase inhibitors and a total of 20 μg of protein was separated by SDS–PAGE electrophoresis and transferred to PVDF membranes (Amersham International, GE Healthcare). Membranes were incubated with blocking solution (5% milk powder in tris-buffered saline-tween 20 (TBST)) for 1 h, then with primary antibody (in blocking solution) overnight at 4 °C. After several washes in TBST, membranes were incubated with horseradish peroxidase (HRP)-conjugated secondary antibodies for 1 h at room temperature (RT) in blocking solution. Membranes were incubated with ECL western-blotting substrate (Amersham International, GE Healthcare) and imaged in a Chemidoc XRS system or ChemiDOC (Bio-Rad Laboratories).

### Tissue histology and immunostaining

For H&E staining, tissues were fixed in 4% paraformaldehyde overnight at room temperature (RT) and followed by dehydration in 70% ethanol. tissues were embedded in paraffin, sectioned at a thickness of 5 μm, and stained with H&E following the standard protocol. COS-7 cells transfected with CSF1R-GFP were washed in PBS and incubated with parvalbumin (200 ng/ml) for 30 min on ice. Sodium azide and Pitstop 2 (ab120687) were added to block receptor internalization. Cells were then fixed with 4% paraformaldehyde for 15 min and blocked in 5% BSA for 30 min at RT and subsequently incubated with parvalbumin antibody (1:1000) in 5% BSA for 2 h at RT. After incubating with a secondary antibody against rabbit IgG conjugated to CY3, cells were co-stained with DAPI (Sigma), and mounted with Antifade reagent (Invitrogen) for fluorescence microscopy. For oil red o staining, tissue slices fixed with 4% paraformaldehyde and embedded with OCT (TissueTek) were rapidly frozen using liquid nitrogen and stored at −80 °C. Sections in 10 μm thick were incubated with 0.3% oil red O solution (in 60% isopropanol) at RT for 10 min and followed by microscope analysis. Images were captured using a Leica DFC 5400 digital camera and were processed using Leica Application Suite v4.13

### Surface plasmon resonance

The equilibrium-binding constant (KD) of parvalbumin and CSF1R was determined by Open SPR (Nicoya, Canada). Briefly, CSF1R (30 µg/ml) was covalently immobilized on COOH-sensor chips (Nicoya, Canada) by the EDC/NHS chemistry. Then, parvalbumin was continuously diluted into several different concentrations using the running buffer and injected into the chip from low to high concentrations. Meanwhile, BSA was used as a negative control. In each cycle, a 250 µl sample has flowed through the chip for 5 min at a constant flow rate of 20 µl/min. After detection, 0.02% SDS was added to dissociate the peptides from target protein. Finally, the kinetic parameters of the binding reactions were calculated and analyzed by Trace Drawer software (Ridgeview Instruments AB, The Kingdom of Sweden).

### Indirect calorimetry

Food intake, oxygen consumption (VO2), carbon dioxide production (VCO2) and locomotor activity were measured in a subgroup of mice using a Comprehensive Lab Animal Monitoring System (Columbus Instruments, Columbus, OH) and VitalView System (MiniMitter, Bend, Oregon, USA). In brief, male mice housed individually with free access to food and water were acclimatized to the metabolic cages for 24 h prior to a 48 h period of automated recordings every 15 min. Sample air from individual cages was passed through sensors to determine O2 and CO2 content by an open-circuit Oxymax.

### Flow cytometry analysis

scWAT were removed, minced in phosphate-buffered saline (PBS) containing 10 mM CaCl_2_, digested at 37 °C for 60–90 min in DMEM containing 10 mM CaCl_2_, 1 mg/ml collagenase type I (Sigma-Aldrich, SCR103), filtered through a 70-μm strainer and centrifuged at 700 g for 5 min. SVF pellets were collected, and resuspended in red-blood-cell lysis buffer (eBioscience RBC Lysis Buffer) before further analysis. To isolate macrophages, SVF pellets were washed twice with PBS and once with FACS staining buffer and stained with F4/80 APC (Biolegend, 123115, 1:200), CD11b PE (Biolegend, 101207, 1:200), CD11C Percp (Biolegend, 117325, 1:100), CD206 FITC (Biolegend, 141703, 1:200) on ice for 1 h in dark. After staining, cells were washed twice with staining buffer, suspended in flow cytometry buffer (PBS with 0.5% BSA and 2 mM EDTA) and subjected to BD LSRFortessa Cell Analyzer (BD Biosciences). The gating strategy for isolated ATM from SVF were according to the previous described^73^. Data were analyzed using FlowJo software version X.0.7 (Tree Star, Inc.) Gating strategy (Fig. s8).

### Immunoprecipitations and kinase assays

Mouse BMDMs were treated with PBS, MCSF, or MSCF combined with parvalbumin for 24 h. Then cells were resuspended on ice with intermediated-efficacy RIPA buffer for 20 min. A total of 4 μg of the anti-Rictor or anti-mTOR antibodies were added to the cleared cellular lysates and incubated with rotation for 90 min. In total 25 μl of protein G-sepharose was then added and the incubation continued for 1 h. Immunoprecipitated captured with protein G-sepharose were washed four times with ice-cold lysis buffer and once with the rictor-mtor kinase buffer (25 mM, Hepes pH 7.5, 100 mM potassium acetate, 1 mM MgCl2). For kinase reaction, immunoprecipitates were incubated in a final volume of 15 μl for 60 min at 37 °C in the rictor-mTOR kinase buffer containing 500 ng recombinant inactive AKT and 500 μM ATP. The reaction was stopped by the addition of 200 μl sample buffer. The samples were analyzed by immunoblotting.

### Molecular docking and parvalbumin blocker preparation

The structure of CSF1R was retrieved from RCSB PDB (PDB ID: 4EXP)^74^ and the structure of parvalbumin was modeled by SWISS-MODEL sever^75^. Molecular docking of CSF1R and parvalbumin was performed using Hex software^76^ by setting CSF1R and parvalbumin as receptor and ligand respectively.

The molecule docking method was used to identify the binding surface of parvalbumin-CSF1R complex. The residues R142, E143, R146, and R150 in M141-Y154 of CSF1R were found to be necessary for the binding of parvalbumin-CSF1R. The M141-Y154 region of CSF1R (MREGGRQVLRKTVY) was then synthesized as a recombinant peptide and was used as a parvalbumin blocker to antagonize parvalbumin–CSF1R interaction. The parvalbumin blocker (HPLC purity > 97%) was synthesized by GenScript USA Inc.

### Quantification and statistical analyses

Experimental data were recorded in Microsoft Excel for Mac, version 16.35. All data are shown as mean ± Standard Error of the Mean (SEM) unless otherwise specified. All datasets were tested for normal distribution using Shapiro-Wilk tests. Skewed data were log-transformed. Results were evaluated using unpaired (2-sided) student’s *t-test*, Welch’s two-sample *t* test and one- or two-way analysis of variance (ANOVA). The assessment of thermogenesis and indirect calorimetry were analyzed of the data with ANCOVA using weight as a covariable was reported^77^. For correlation analyses, Pearson’s correlation coefficient was used to analyze the correlation between serum parvalbumin levels and BMI, Waistline, Hipline, and FBG. Statistically significant differences were considered to be *p* < 0.05. The relevant statistical methods for each panel were detailed in the figure legend. All statistical analyses were performed using GraphPad or SPSS 22.0. For the histology analysis and western blot analysis, each experiment was repeated three times independently with similar results.

## Supplementary information

Supplementary Information

Description of additional Supplementary File

Supplementary Data1

## Data Availability

The mass spectrometry proteomics data have been deposited to the ProteomeXchange Consortium via the PRIDE partner repository with the dataset identifier ProteomeXchange: PXD033582. The sequencing data used in this study is from GEO database under accession code GSE 9103. Uniprot-SwissProt database [https://www.uniprot.org/uniprot/?query=reviewed%3Ayes%20taxonomy%3A10090&columns=id%2Centry%20name%2Creviewed%2Cprotein%20names%2Cgenes%2Corganism%2Clength] was used to labeled protein information. Source data for Figs. 1–7 and Extended Data Figs. 1–8 containing raw data for all experiments are provided with this paper. Source data are provided with this paper.

## Source data

Source Data
