# Supplementary material for: Targeting parvalbumin promotes M2 macrophage polarization and energy expenditure in mice
Source: Nat Commun. 2022 Jun 8;13:3301. doi: 10.1038/s41467-022-30757-y (PMC9177846; doi:10.1038/s41467-022-30757-y)
Supplement: Supplementary file 1 — Supplementary Information [file 41467_2022_30757_MOESM1_ESM.pdf]

Supplementary information

Targeting parvalbumin promotes M2 macrophage polarization and energy expenditure in mice

Shaojian Lin, Anke Zhang, Ling Yuan, Yufan Wang, Chuan Zhang, Junkun Jiang, Houshi Xu, Huiwen Yuan, Hui Yao, Qianying Zhang, Yong Zhang, Meiqing Lou, Ping Wang, Zhen-ning Zhang, Bing Luan

Supplementary Figures and Tables

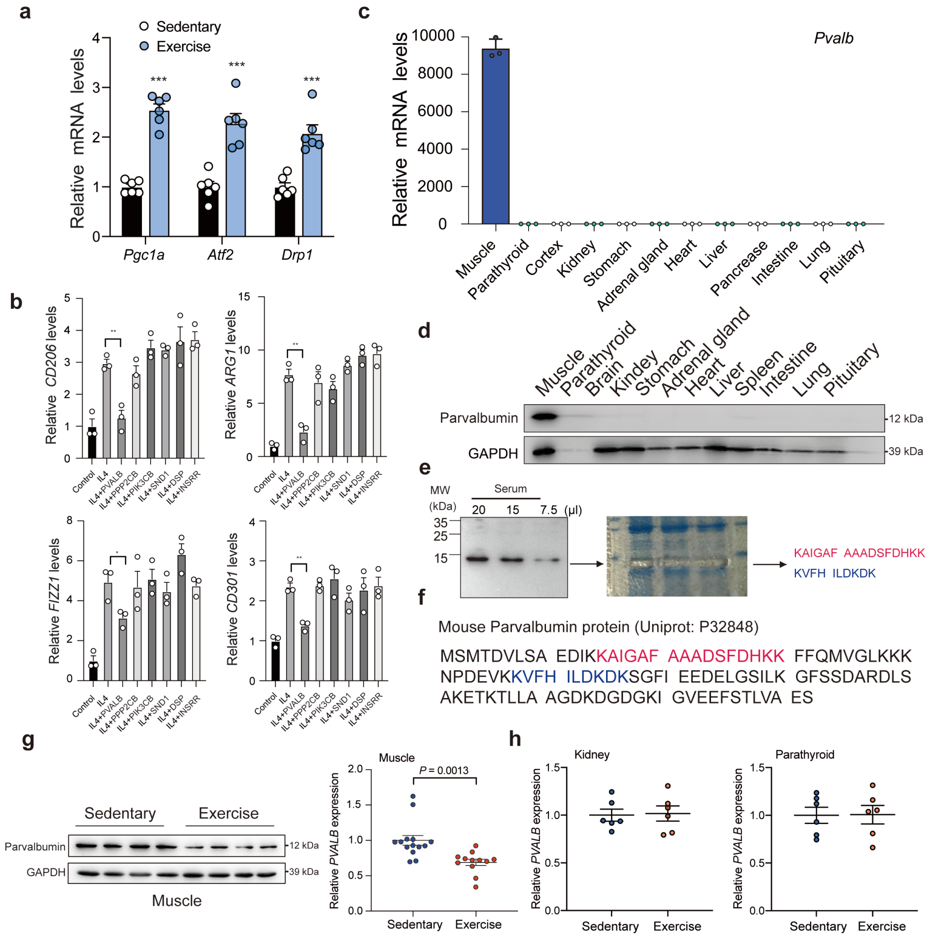

Supplementary Figure 1. Validation of parvalbumin detection from serum.

(a) Relative mRNA expression of mitochondrial adaption-associated genes (*Pgc1α, Atf2, and Drp1*) in muscle from exercised or sedentary mice (*n* = 6 per group). p value: <0.0001, 0.0002, 0.0002. **(b)** Representative M2 macrophage marker gene expression in PBMC treated with IL4 combined with indicated human recombinant proteins (parvalbumin, PPP2CB, PIK3CB, SND1, DSP, and INSRR) (*n* = 3 per group). p value: 0.0299, 0.0033, 0.0012, 0.0017. **(c)** Relative *Pvalb* mRNA expression in indicated tissues of C57BL6/N mice (*n* = 3 per group). Values are normalized to *GAPDH.* **(d)** Parvalbumin protein expression in indicated tissues of C57BL6/N mice. **(e)** Immunoblot of the indicated volume of mouse serum with anti-parvalbumin (left). Immunolabeled bands were used to guide excision of the corresponding bands from the other half of the SDS-PAGE gel that had not been electroblotted (right). Band excision was guided by overlaying the unstained gel onto an image of the developed immunoblot (right). Excised bands were subjected to in-gel tryptic digestion followed by mass spectrometry analysis, as described in the Methods. **(f)** The full-length amino acid sequence of parvalbumin. Peptides identified by mass spectrometry in excised bans are highlighted in pink and blue. **(g)** Parvalbumin protein and mRNA expression in muscle from exercised or sedentary mice (Sedentary, *n* = 14; Exercise, n = 12). **(h)** Parvalbumin mRNA expression in kidney and parathyroid from exercised or sedentary mice (*n* = 3 per group). Data are represented as mean ± SEM. *P* values were determined by unpaired two-tailed Student's t-test **(a, b, g, and h)**. *p < 0.05, **p < 0.01, ***p < 0.001. Source data are provided as a Source Data file.

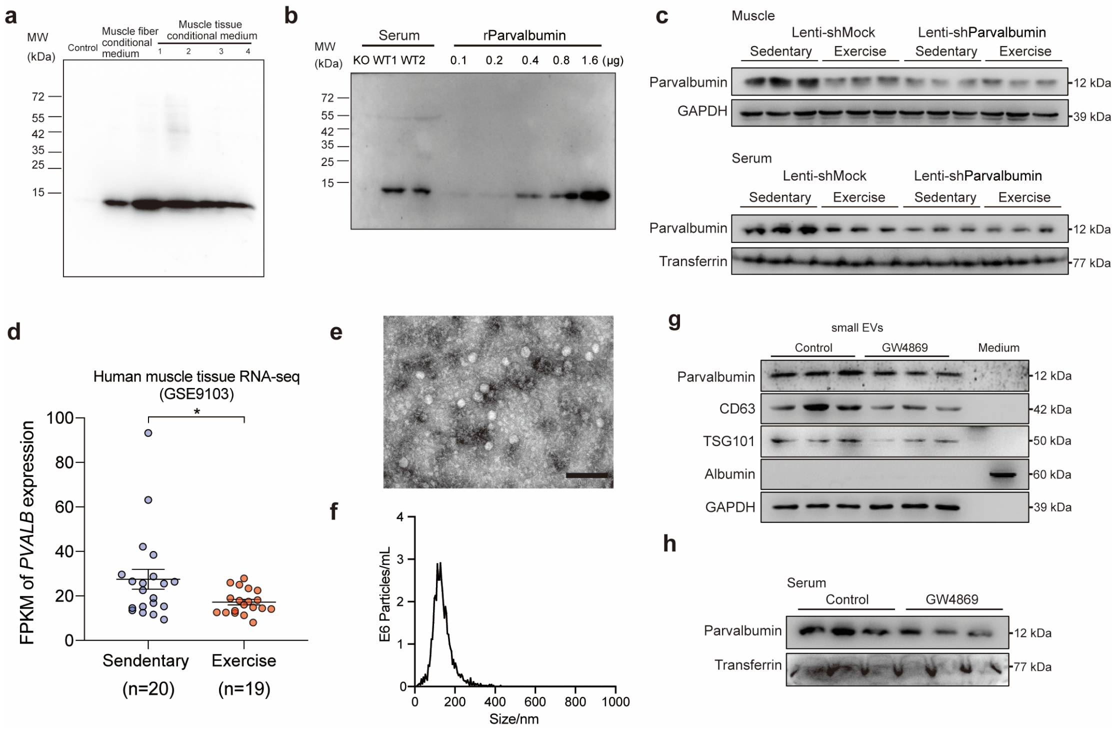

Supplementary Figure 2. Validation of parvalbumin secretion from muscle.

(a) Representative immunoblots of secreted parvalbumin in conditional medium from control, muscle fiber and muscle tissues of four mice. **(b)** Representative immunoblots of serum parvalbumin from WT and parvalbumin-KO mice with anti-parvalbumin. Different doses of rparvalbumin were loaded. **(c)** Abundance of parvalbumin in muscle and serum of mice with local injection of shMock-GFP and shParvalbumin-GFP lentivirus. **(d)** Comparison of parvalbumin expression level in human muscle between sedentary and exercise group. The data was extracted from the GEO dataset (GSE 9103). p value: 0.0372. **(e)** Representative electron microscopy image of muscle-derived small EVs. Bar: 100 nm. **(f)** The EVs particle size and concentration were measured by nanoparticle tracking analysis (NTA). **(g)** Abundance of parvalbumin in muscle-derived exosome and (**h**) serum of mice treated with GW4869. Data are represented as mean ± SEM. *P* values were determined by unpaired two-tailed Student's t-test **(d)**. *p < 0.05. Source data are provided as a Source Data file.

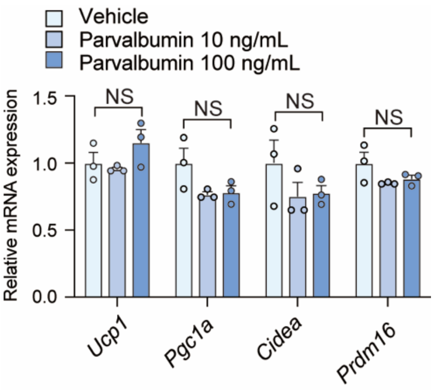

Supplementary Figure 3. No detectable effect of endogenous parvalbumin on the regulation of thermogenic genes

Representative thermogenic gene expression in SVF cells exposed to indicated dose of rparvalbumin (*n* = 3 per group). *P* values were determined by unpaired two-tailed Student's t-test. Ns, no significance. Data are represented as mean ± SEM. *P* values were determined by unpaired two-tailed Student's t-test. Source data are provided as a Source Data file.

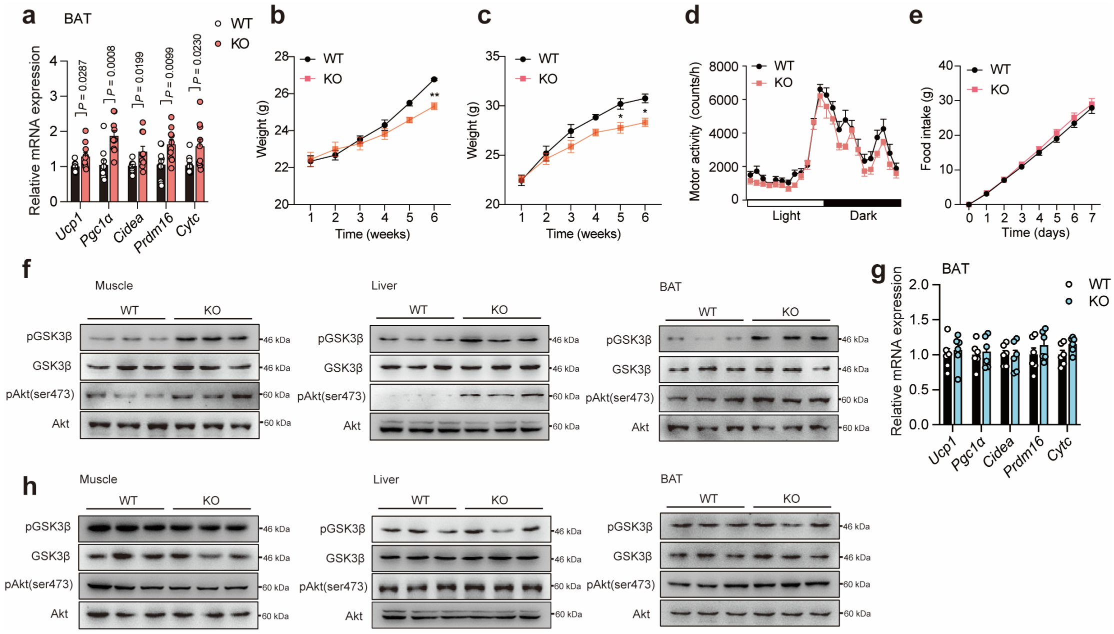

Supplementary Figure 4. Effect of parvalbumin on thermogenesis and insulin signaling under room temperature and thermoneutrality.

(a) Representative thermogenic gene expression in BAT of WT and parvalbumin KO mice under room temperature (*n* = 10 per group). **(b and c)** Change of body weight of mice on regular chow (b) (p value: 0.0139, 0.0139) and high-fat diet (c) (*n* = 4 per group) (p value: 0.0029). **(d)** Locomotor activity of WT and parvalbumin KO mice (*n* = 6 per group). **(e)** Cumulative feed intake of WT and parvalbumin KO mice fed on RD (*n* = 10 per group). **(f)** Representative immunoblots showing effects of parvalbumin KO on insulin signaling in muscle, liver and BAT tissues under room temperature. **(g)** Representative thermogenic gene expression in BAT of WT and parvalbumin-KO mice under thermoneutrality condition (n = 6 per group). **(h)** Representative immunoblots showing effects of parvalbumin KO on insulin signaling in muscle, liver and BAT tissues under thermoneutrality condition. Data are represented as mean ± SEM. *P* values were determined by unpaired two-tailed Student's t-test **(a, and g)** and two-way ANOVA with Tukey's post hoc tests **(b-e).** *p < 0.05, **p < 0.01. Source data are provided as a Source Data file.

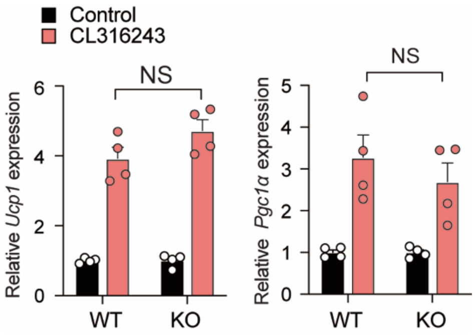

Supplementary Figure 5. *Ucp1* and *Pgc1a* expression in SVF of scWAT from WT and parvalbumin-KO mice treated with CL316243

Data are represented as mean ± SEM. *P* values were determined by unpaired two-tailed Student's t-test. Ns, no significance. Source data are provided as a Source Data file.

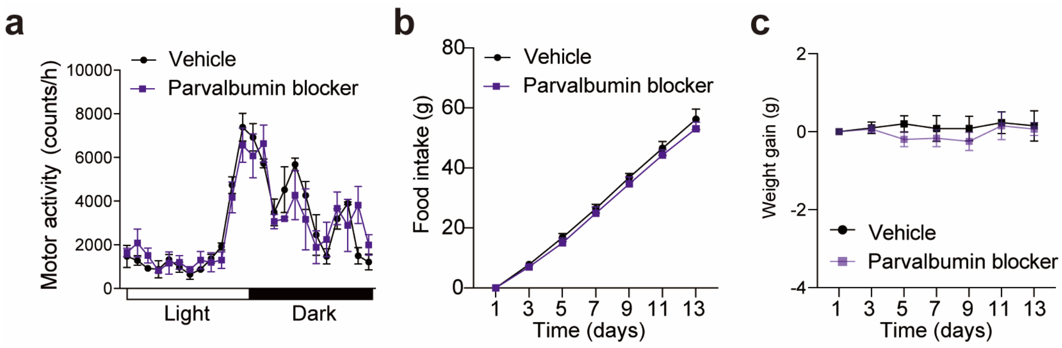

Supplementary Figure 6. No effect of parvalbumin blocker on food intake and motor activity. **(a)** Locomotor activity of mice treated with parvalbumin blocker (*n* = 3 per group). **(b)** cumulative feed intake of mice treated with parvalbumin blocker (*n* = 6 per group). **(c)** body weight gain of mice treated with parvalbumin blocker under thermoneutrality (*n* = 4 per group). Data are represented as mean ± SEM. *P* values were determined by two-way ANOVA with Tukey's post hoc tests **(a-c).** Source data are provided as a Source Data file.

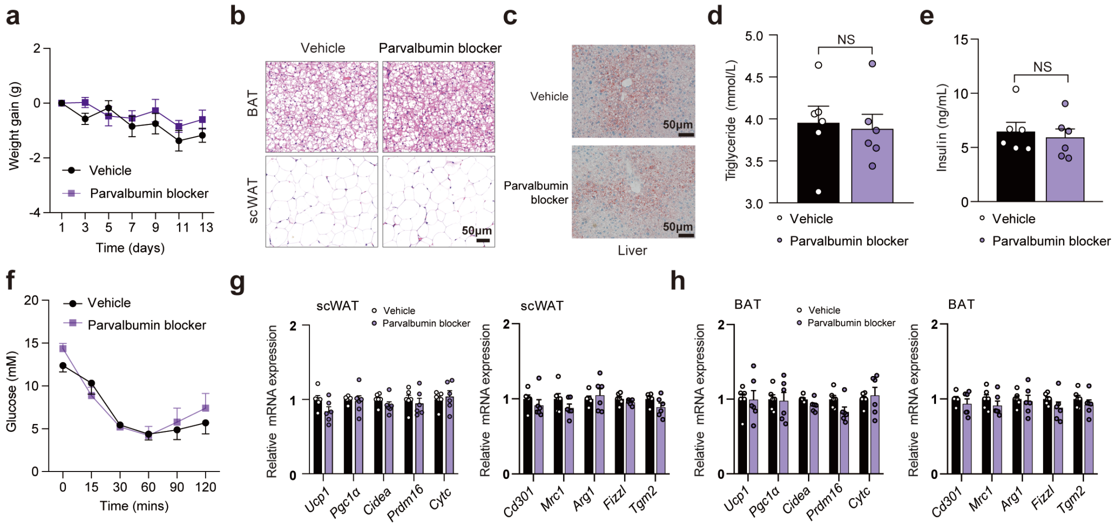

Supplementary Figure 7. Parvalbumin-dependent effect of parvalbumin blocker in HFD-induced obesity.

(a to h) HFD-fed parvalbumin KO mice were injected daily with vehicle (*n* = 6 per group) or parvalbumin blocker (5 mg kg-1, *n* = 6 per group) for 2 weeks. The weight gain (**a**), representative images of H&E staining of BAT and scWAT (**b**), representative images of Oil O Red staining of liver (**c**), circulating triglycerides (**d**), serum insulin **(e)**, insulin tolerance test (**f**), and representative thermogenic and M2 macrophage marker gene expression in scWAT and BAT (**g** and **h**) of vehicle or parvalbumin blocker injected mice were determined (*n* = 6 per group). Scale bar = 50 μm. Data are represented as mean ± SEM. *P* values were determined by two-way ANOVA with Tukey's post hoc tests **(a and f),** and unpaired two-tailed Student's t-test (**d, e, g and h**). Source data are provided as a Source Data file.

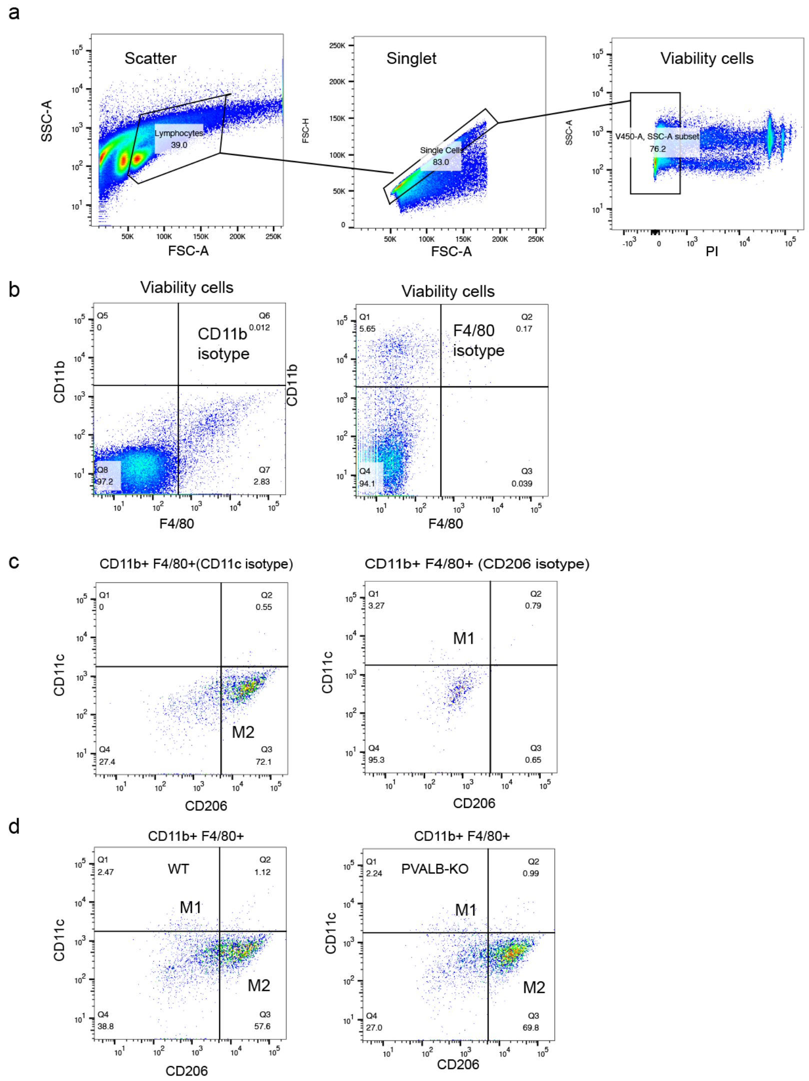

Supplementary Figure 8. The gating strategy for isolated ATM from SVF

(a-c) Gating strategy to sort singlet and viability cells from WT and parvalbumin KO mice (a). Modified FMO controls incorporating isotype antibodies for F4/80 and CD11b were used to align quadrant gates (b). Modified FMO controls incorporating isotype antibodies for F4/80+ CD11b + following cd11c+ and F4/80+ CD11b + following CD206+ were used to align quadrant gates (c). **(d)** Based on a-c gating strategy, comparison of proportion of CD206+ (M2) or CD11c+ (M1) from the of F4/80+CD11b+ ATMs of WT and parvalbumin-KO mice.

Supplementary Table 1. Clinical data of lean and obese individuals.

ID 1-58 were obese patients, and 59-81 were lean individuals.

ID	Age	BMI	Lean/ Obesity	waistline (cm)	hipline (cm)	FBG (mmol/L)
**1**	43	29.03	Obesity	96.00	99.00	8.09
**2**	25	29.59	Obesity	108.00	103.00	6.03
**3**	47	36.77	Obesity	96.00	99.00	9.00
**4**	33	36.30	Obesity	103.00	105.00	12.70
**5**	38	34.66	Obesity	96.00	98.00	8.00
**6**	46	35.27	Obesity	105.00	106.00	15.21
**7**	39	34.48	Obesity	100.00	102.00	15.21
**8**	31	38.76	Obesity	108.00	110.00	7.22
**9**	49	37.76	Obesity	121.00	115.00	7.02
**10**	41	30.42	Obesity	105.00	104.00	12.78
**11**	32	30.12	Obesity	95.00	98.00	12.52
**12**	35	31.51	Obesity	98.00	98.00	5.79
**13**	39	30.40	Obesity	99.00	103.00	8.26
**14**	33	37.40	Obesity	120.00	119.00	9.23
**15**	34	34.50	Obesity	102.00	105.00	10.49
**16**	49	34.40	Obesity	105.00	108.00	7.73
**17**	40	32.90	Obesity	103.00	105.00	8.69
**18**	37	32.90	Obesity	110.00	105.00	8.08
**19**	52	31.00	Obesity	103.00	105.00	7.56
**20**	25	36.20	Obesity	117.00	115.00	6.19
**21**	50	40.30	Obesity	135.00	136.00	10.44
**22**	71	31.40	Obesity	106.00	107.00	5.49
**23**	55	31.30	Obesity	103.00	100.00	9.07
**24**	39	30.50	Obesity	105.00	101.00	13.88
**25**	31	31.50	Obesity	103.00	100.00	5.57
**26**	50	32.20	Obesity	112.00	105.00	6.73
**27**	39	30.40	Obesity	99.00	103.00	8.26
**28**	47	33.80	Obesity	104.00	108.00	6.71
**29**	31	31.30	Obesity	100.00	106.00	10.00
**30**	33	36.30	Obesity	103.00	105.00	7.43
**31**	46	35.27	Obesity	105.00	106.00	15.21
**32**	55	34.11	Obesity	102.00	105.00	9.63
**33**	27	33.60	Obesity	111.00	112.00	3.99
**34**	44	31.80	Obesity	104.00	101.00	9.09
**35**	48	30.50	Obesity	105.00	110.00	7.80
**36**	44	31.00	Obesity	103.00	106.00	6.29
**37**	50	31.70	Obesity	110.00	108.00	8.02
**38**	26	31.40	Obesity	101.00	108.00	7.47
**39**	33	30.80	Obesity	107.00	109.00	6.11
**40**	46	30.40	Obesity	95.00	100.00	6.72

**41**	46	33.50	Obesity	119.00	118.00	6.07
**42**	38	31.10	Obesity	103.00	105.00	4.70
**43**	36	32.20	Obesity	98.00	100.00	10.30
**44**	53	30.80	Obesity	101.00	107.00	6.62
**45**	48	31.10	Obesity	104.00	103.00	9.19
**46**	56	32.20	Obesity	109.00	107.00	9.65
**47**	51	32.10	Obesity	104.00	101.00	9.60
**48**	47	30.70	Obesity	103.00	102.00	5.48
**49**	57	31.40	Obesity	115.00	110.00	6.90
**50**	50	31.30	Obesity	104.00	115.00	8.78
**51**	36	31.20	Obesity	102.00	105.00	5.76
**52**	30	31.00	Obesity	100.00	104.00	8.13
**53**	39	30.60	Obesity	98.00	101.00	3.63
**54**	29	33.50	Obesity	108.00	112.00	6.03
**55**	47	31.10	Obesity	102.00	106.00	8.69
**56**	55	30.40	Obesity	102.00	103.00	8.90
**57**	25	33.00	Obesity	112.00	107.00	10.26
**58**	25	31.00	Obesity	105.00	105.00	4.42
**59**	39	24.20	Lean	76.00	85.00	3.77
**60**	45	21.20	Lean	71.00	89.00	4.09
**61**	43	23.00	Lean	83.00	94.00	5.18
**62**	29	21.50	Lean	72.00	96.00	4.59
**63**	38	24.10	Lean	86.00	94.00	5.07
**64**	44	29.00	Lean	90.00	85.00	5.26
**65**	41	24.30	Lean	94.00	89.00	5.03
**66**	47	22.73	Lean	90.00	94.00	4.84
**67**	48	24.60	Lean	92.00	96.00	5.10
**68**	37	21.30	Lean	80.00	94.00	5.48
**69**	32	24.90	Lean	84.00	85.00	4.78
**70**	41	24.17	Lean	83.00	89.00	4.78
**71**	35	23.15	Lean	93.00	94.00	5.17
**72**	50	21.97	Lean	76.00	96.00	5.26
**73**	28	23.94	Lean	71.00	94.00	4.46
**74**	30	22.81	Lean	83.00	89.00	4.99
**75**	27	22.50	Lean	72.00	94.00	4.28
**76**	32	24.58	Lean	86.00	96.00	4.15
**77**	27	23.36	Lean	90.00	94.00	3.95
**78**	57	23.40	Lean	94.00	101.00	5.10
**79**	35	22.53	Lean	90.00	98.00	4.83
**80**	31	20.69	Lean	92.00	98.00	4.87
**81**	38	24.99	Lean	84.00	100.00	4.82

Supplementary Table 2. List of antibodies used.

Name	brand	catalogue	dilutions	Application
**Transferrin**	Abbkine	ABM40235	1:1000	WB
**UCP1**	Abcam	ab155117	1:2000	WB
**pPKC**	Abcam	ab180848	1:2000	WB
**PKC**	Abcam	ab179522	1:2000	WB
**Rictor**	Abcam	ab70374	1:100	IP
**Parvalbumin**	ABclonal	A13538	1:1000	WB
**F4/80 APC**	Biolegend	123115	1:200	FACS
**CD11b PE**	Biolegend	101207	1:200	FACS
**CD11C Percp**	Biolegend	117325	1:100	FACS
**CD206 FITC**	Biolegend	141703	1:200	FACS
**pSTST6**	Cell Signaling Tech	56554	1:2000	WB
**STAT6**	Cell Signaling Tech	9362	1:2000	WB
**pAKT (Ser473)**	Cell Signaling Tech	4060	1:2000	WB
**pAKT (Thr308)**	Cell Signaling Tech	13038	1:2000	WB
**AKT**	Cell Signaling Tech	2920	1:2000	WB
**pERK**	Cell Signaling Tech	4370	1:2000	WB
**ERK**	Cell Signaling Tech	4695	1:2000	WB
**p4EBP1**	Cell Signaling Tech	2855	1:2000	WB
**4EBP1**	Cell Signaling Tech	9452	1:2000	WB
**HRP-conjugated goat anti-Rabbit IgG**	Cell Signaling Tech	7074	1:10000	WB
**pGSK-3β**	Cell Signaling Tech	5558	1:2000	WB
**GSK-3β**	Cell Signaling Tech	12456	1:2000	WB
**mTOR**	Cell Signaling Tech	2983	1:100	IP
**GFP**	Proteintech	50430-2-AP	1:1000	WB
**GAPDH**	Santa Cruz	sc32233	1:1000	WB
**ACTB**	Sigma Aldrich	A3854	1:10000	WB
**Flag**	Sigma-Aldrich	F7425	1:10000	WB

Supplementary Table 3. Primers for RT-PCR.

Genes	Forward primers 5' to 3'	Reversed primers 5' to 3'
**Mouse *Ucp1***	AGGCTTCCAGTACCATTAGGT	CTGAGTGAGGCAAAGCTGATTT
**Mouse *Pgc1a***	CCCTGCCATTGTTAAGACC	TGCTGCTGTTCCTGTTTTC
		doi: 10.1371/journal.pone.0112495
**Mouse *Cidea***	TGCTCTTCTGTATCGCCCAGT	GCCGTGTTAAGGAATCTGCTG
		doi: 10.1016/j.physbeh.2019.01.004
**Mouse *Prdm16***	CCACCAGCGAGGACTTCAC	GGAGGACTCTCGTAGCTCGAA
**Mouse *Cytc***	ACAAGAAGACTCAAATGTGTTTCAGTTT	TGCACTGTCAAGAATAGACAGTTGC
		doi:10.1074/jbc.M115.705822
**Mouse *Adipoq***	TGTTCCTCTTAATCCTGCCCA	CCAACCTGCACAAGTTCCCTT
**Mouse *Ear2***	CCTGTAACCCCAGAACTCCA	CAGATGAGCAAAGGTGCAAA
		doi: 10.1080/21623945.2015.1040612
**Mouse *Ap2***	ATGGAGGAGAGTGAGTACGAGT	CATTCTGAGGCCCTGTAACCA
		doi：10.4049/jimmunol.1401288
**Mouse *Fabp4***	AAGGTGAAGAGCATCATAACCCT	TCACGCCTTTCATAACACATTCC
**Mouse *Cd301***	CAGCTTGCTCCCCTCTACCT	TCCAACGACCATCGTAAGAAAAG
		Self validation
**Mouse *Cd206***	CTCTGTTCAGCTATTGGACGC	CGGAATTTCTGGGATTCAGCTTC
**Mouse *Arg1***	CTCCAAGCCAAAGTCCTTAGAG	AGGAGCTGTCATTAGGGACATC
**Mouse *Fizz1***	CTGCCCTGCTGGGATGACT	CATCATATCAAAGCTGGGTTCTCC、
		doi：10.1152/ajpcell.00370.2015
**Mouse *Tgm2***	GACAATGTGGAGGAGGGATCT	CTCTAGGCTGAGACGGTACAG
**Mouse *Rantes***	GCTGCTTTGCCTACCTCTCC	TCGAGTGACAAACACGACTGC
**Mouse *Mcp1***	CCACTCACCTGCTGCTACTCA	TGGTGATCCTCTTGTAGCTCTCC
		doi：10.1016/j.mce.2014.12.017
**Mouse *Tnfa***	ACGGCATGGATCTCAAAGAC	AGATAGCAAATCGGCTGACG
		doi：10.1016/j.heliyon.2019.e01195
**Mouse *Il4***	GGTCACAGGAGAAGGGACGCC	TGCGAAGCACCTTGGAAGCCC
**Mouse *Il5***	AGGCTTCCTGTCCCTACTCA	CCCCCACGGACAGTTTGATT
		doi：10.1038/s41598-020-60089-0
**Mouse *Il13***	ACCGAAATGTTGATAGCGACAG	ACAATGCTCTGACAAATGCGTA
		doi：10.1016/j.nutres.2017.10.007
**Human *CD301***	AGCAACTTCACCTCAAACACTG	AGATGCTATCGTTTCTTCCAAGC
**Human *MRC1***	TCCGGGTGCTGTTCTCCTA	CCAGTCTGTTTTTGATGGCACT
**Human *FIZZ1***	CCGTCCTCTTGCCTCCTTC	CTTTTGACACTAGCACACGAGA
**Human *ARG1***	GTGGAAACTTGCATGGACAAC	AATCCTGGCACATCGGGAATC

Red: Primers were validated by Primerbank; Green: Primers were validated by previous studies; Black: Self validation.
