# Supplementary material for: Targeting parvalbumin promotes M2 macrophage polarization and energy expenditure in mice
Source: Nat Commun. 2022 Jun 8;13:3301. doi: 10.1038/s41467-022-30757-y (PMC9177846; doi:10.1038/s41467-022-30757-y)
Supplement: Supplementary file 2 — Description of additional Supplementary File [file 41467_2022_30757_MOESM2_ESM.pdf]

Description of additional supplementary data files

Supplementary Data 1: Quantitative proteomics on serum samples from sedentary and exercised mice.
